# Long‐term community shifts driven by local extinction of an iconic foundation species following an extreme marine heatwave

**DOI:** 10.1002/ece3.10235

**Published:** 2023-06-26

**Authors:** Shinae Montie, Mads S. Thomsen

**Affiliations:** ^1^ Marine Ecology Research Group, School of Biological Sciences University of Canterbury Christchurch New Zealand; ^2^ Department of Ecoscience Aarhus University Roskilde Denmark

**Keywords:** attachment networks, heatwaves, local extinction, seaweed, succession

## Abstract

Gradual ocean warming combined with stronger marine heatwaves (MHWs) can reduce abundances of foundation species that control community structures, biodiversity, and ecosystem functioning. However, few studies have documented long‐term succession trajectories following the more extreme events that cause localized extinctions of foundation species. Here, we documented long‐term successional changes to marine benthic communities in Pile Bay, New Zealand, following the Tasman 2017/18 MHW, which caused localized extinctions of dominant southern bull kelp (*Durvillaea* sp.). Six years on, multiscale annual and seasonal surveys show no sign of *Durvillaea* recolonization. Instead, the invasive annual kelp (*Undaria pinnatifida*), rapidly colonized areas previously dominated by *Durvillaea*, followed by large changes to the understory community, as *Durvillaea* holdfasts and encrusting coralline algae were replaced by coralline turf. Between 3 and 6 years after the total loss of *Durvillaea*, smaller native fucoids colonized in high densities. Although *Undaria* initially colonized plots throughout *Durvillaea*'s tidal range, later in the succession *Undaria* only retained dominance in the lower intertidal zone and only in spring. Ultimately, the tidal zone was slowly replaced by alternative foundation species, composed of different canopy‐forming brown seaweeds that dominated different intertidal elevations, resulting in a net increase in canopy and understory diversity. This study is a rare example of long‐term effects following an extreme MHW that caused extinctions of a locally dominant canopy‐former, but these events and their associated dramatic changes to community structures and biodiversity are expected to become increasingly common as MHWs continue to increase in strength, frequency, and duration.

## Graphical abstract

Global warming and stronger marine heatwaves (MHWs) can in extreme cases cause localized extinctions of the key foundation species that control community structures, biodiversity, and ecosystem functioning. Here, we provide a world‐first case study of dramatic long‐term and detailed successional changes to marine benthic communities after an extreme MHW that caused localized extinctions of the iconic southern bull kelp (*Durvillaea* sp.) that epitomize the rugged wave‐exposed coastlines of New Zealand. Although this study is a very rare example of long‐term community effects following an extreme MHW and local extinctions of foundation species, these types of MHW‐driven ecosystem shifts are expected to become increasingly common in the near future.

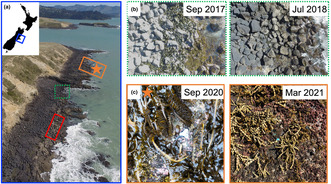

## 1 INTRODUCTION

Recent studies have documented intensification of marine heatwaves (MHWs; Frölicher et al., 2018; Oliver et al., 2019, 2021; Thoral et al., 2022) and their impacts on marine organisms, including competitively dominant foundation species that control community structures (Doney et al., 2012; Smale et al., 2019). Impacts from MHWs on the ecological performance of foundation species has typically been reported on canopy‐forming seagrass (Arias‐Ortiz et al., 2018; Strydom et al., 2020) and different large brown seaweed (Arafeh‐Dalmau et al., 2019; Filbee‐Dexter et al., 2020; Rogers‐Bennett & Catton, 2019; Smale & Wernberg, 2013; Straub et al., 2019; Tait et al., 2021; Thomsen et al., 2019; Wernberg et al., 2016). During these MHWs, temperatures may exceed physiological thermal optimums resulting in decreased ecological performances and lowered species abundances (Filbee‐Dexter et al., 2020; Rogers‐Bennett & Catton, 2019; Smith et al., 2023; Spiecker & Menge, 2022; Suryan et al., 2021; Tait et al., 2021; Whalen et al., 2023). However, a few rare case studies have documented much more conspicuous ecological effects, like when extreme MHWs cause regional extinction of the competitively dominant foundation species (Smale & Wernberg, 2013; Thomsen et al., 2019; Wernberg et al., 2016). It is likely that localized extinctions of competitively dominant foundation species will become more common because MHWs are predicted to become stronger, longer, and more frequent (Oliver et al., 2019). For example, over the last 40 years, most temperate coastal ecosystems, which often are dominated by large brown seaweeds, have experienced strong increases in MHWs (Thoral et al., 2022). More specifically, similar trends have been observed in coastal New Zealand (Montie et al., 2023), a bioregion characterized by a long temperate coastline, high biodiversity, and unique marine communities with many endemic species (Costello et al., 2010). It is therefore of principal importance to document how coastal ecosystems may change in the future following the total loss of competitively dominant foundation species.

The wave‐exposed intertidal and shallow subtidal coastline on New Zealand's South Island is often dominated by the native foundation species, *Durvillaea antarctica*, *D. willana*, and *D. poha* (the two latter species are also endemic), more generally referred to as southern bull kelp. Southern bull kelp can reach 10 m in length, live for 7–8 years, build canopy‐dominated seascapes, control community structure and productivity, and are therefore considered iconic species that epitomize New Zealand's rugged coastline and kelp‐associated fisheries of abalone, butterfish, and crayfish (Fraser et al., 2009; Hay, 1994; Taylor & Schiel, 2005; Thomsen & South, 2019; Velásquez et al., 2020). During the summer of 2017/18, the east coast of the South Island experienced extreme air and sea surface temperatures, corresponding to the strongest MHW on record (Salinger et al., 2019; Thomsen et al., 2019). This extreme climatic event began on 14 November 2017 and lasted 147 days until 9 April 2018 (Salinger et al., 2019). It resulted in rapid extinction of bull kelp at our study area, Pile Bay, Lyttelton Harbour, and at other sites within and around Lyttelton Harbour (Thomsen et al., 2019). This extinction event was followed by immediate colonization of the invasive kelp *Undaria pinnatifida* (hereafter *Undaria*), one of the world's most widely distributed marine invaders that has inhabited Lyttelton Harbour since the early 1990s (Epstein & Smale, 2017; South et al., 2017; Thomsen et al., 2018, 2019).

Here, we provide a rare case study of the long‐term (6 years) successional trajectories after complete loss of southern bull kelp—the locally competitively dominant foundation species—and colonization by different native and invasive foundation species—across spatiotemporal scales. The few existing case studies suggest that replacement of lost foundation species with alternative foundation species is a slow process (Thomsen & South, 2019; Wernberg et al., 2016). Considering bull kelp were extirpated on all reefs in Lyttelton Harbour and nearby (Thomsen et al., 2019), recolonization would rely on drift of separate male and female fronds during a short reproductive period from autumn to early spring (Fraser et al., 2018; Taylor & Schiel, 2003; Velásquez et al., 2020). We therefore hypothesized that bull kelp would not recolonize Pile Bay and that species that typically co‐occur with bull kelp, like encrusting algae, would also decrease in abundance (Thomsen et al., 2019). We also hypothesized that *Undaria* would continue to benefit from reduced competition from bull kelp and that space previously dominated by bull kelp eventually would be colonized by other seaweed that are common on this particular reef, like coralline turf and other native brown seaweeds (South & Thomsen, 2016).

## 2 METHODS

### 2.1 Regional context

Surveys were conducted along the rocky intertidal zone in Pile Bay (−43.615, 172.765), Lyttelton Harbour, Banks Peninsula. Pile Bay is a less wave‐exposed reef compared to typical bull kelp‐dominated reefs. The reef is composed of volcanic rock (Sewell, 1985) and seasonal SST ranges between 9 and 20°C. However, during the Tasman 2017/18 MHW, SST increased to more than 23°C (Salinger et al., 2019; Thomsen et al., 2019). This was the strongest MHW event recorded over the 38 years of existing satellite data, with maximum and cumulative intensities two times greater than any recorded event since 1980 (Thomsen et al., 2019). The focal foundation species of this study, southern bull kelp, are adapted to wave‐exposed cool, clear, nutrient‐rich waters (Hay, 1994; Velásquez et al., 2020), potentially making them vulnerable to anthropogenic stressors like warming or decreased water clarity. The pre‐MHW bull kelp population in Pile Bay all had wide blades, a yellow‐brownish tint, and were relatively short (<5 m), suggesting that they were dominated by the endemic *D. poha* (and not *D. antarctica*; Fraser et al., 2012; Velásquez et al., 2020).

### 2.2 Changes to dominant seaweed on the reef scale (drone images)

To test whether the Tasman MHW affected the general distribution patterns of intertidal canopy‐forming seaweed in Pile Bay, large‐scale drone surveys were conducted before the MHW in August 2017 with an Advanced Phantom 3 equipped with a 12 MP, 1/2.3″ CMOS sensor and FOV 94° 20 mm lens with an image size of 4000 × 3000 and ground sample distance of 0.43 cm/pixel. Surveys were repeated annually until September 2022 with a Mavic Mini 2 equipped with a 12 MP, 1/2.3″ CMOS sensor and FOV 84° 24 mm lens with an image size of 4000 × 3000 and ground sample distance of 0.36 cm/pixel. Geotagged RGB images were taken at an altitude of 10 m during spring tides (0.1–0.3 m above lowest astronomical tide [LAT]), where each image covered c. 95 m^2^. For each image, percent cover of large canopy‐forming algal species was visually estimated using a superimposed grid of 100 cells and separated into the low, mid, and high intertidal zones, based on cover of water and bare substrate (cover of water between 10% and 40% = low zone, <10% water and <50% bare substrate = mid zone and 0% water and >50% bare substrate = high zone). Changes to community structure and percent cover of the dominant canopy‐forming seaweed were analyzed with two‐way permutational multivariate analyses of variance (PERMANOVA) using Bray–Curtis dissimilarity coefficients for multivariate community data (see Appendix S1 for results) and Euclidian distance for univariate taxa responses. The two fixed factors included year (2017 pre‐MHW, 2018 1‐year, 2019 2‐year, 2020 3‐year, 2021 4‐year, and 2022 5‐year post‐MHW) and elevation (low, medium, and high). Data could not be transformed to achieve homogeneous variances, so results should be interpreted with caution. We therefore adjusted alpha to .01 and acknowledge that significant effects may alternatively have been caused by heterogeneous variances (Underwood et al., 1997). However, because we were interested in evaluating interaction effects and assessing relative importance of crossed test factors (Levine & Hullett, 2002), biased factorial PERMANOVAs were preferred over nonparametric ranked tests that do not address interaction terms or relative importance of test factors (but still require similar distributions). Furthermore, PERMANOVA approaches are generally robust to minor heteroscedasticity for balanced designs (Quinn & Keough, 2002; Underwood et al., 1997). A similarity of percentages breakdown (SIMPER) was used to assess the contribution of individual species to community dissimilarity between years and elevations.

### 2.3 Common species in pre‐heatwave bull kelp forest (understory photographs)

**FIGURE 1:**
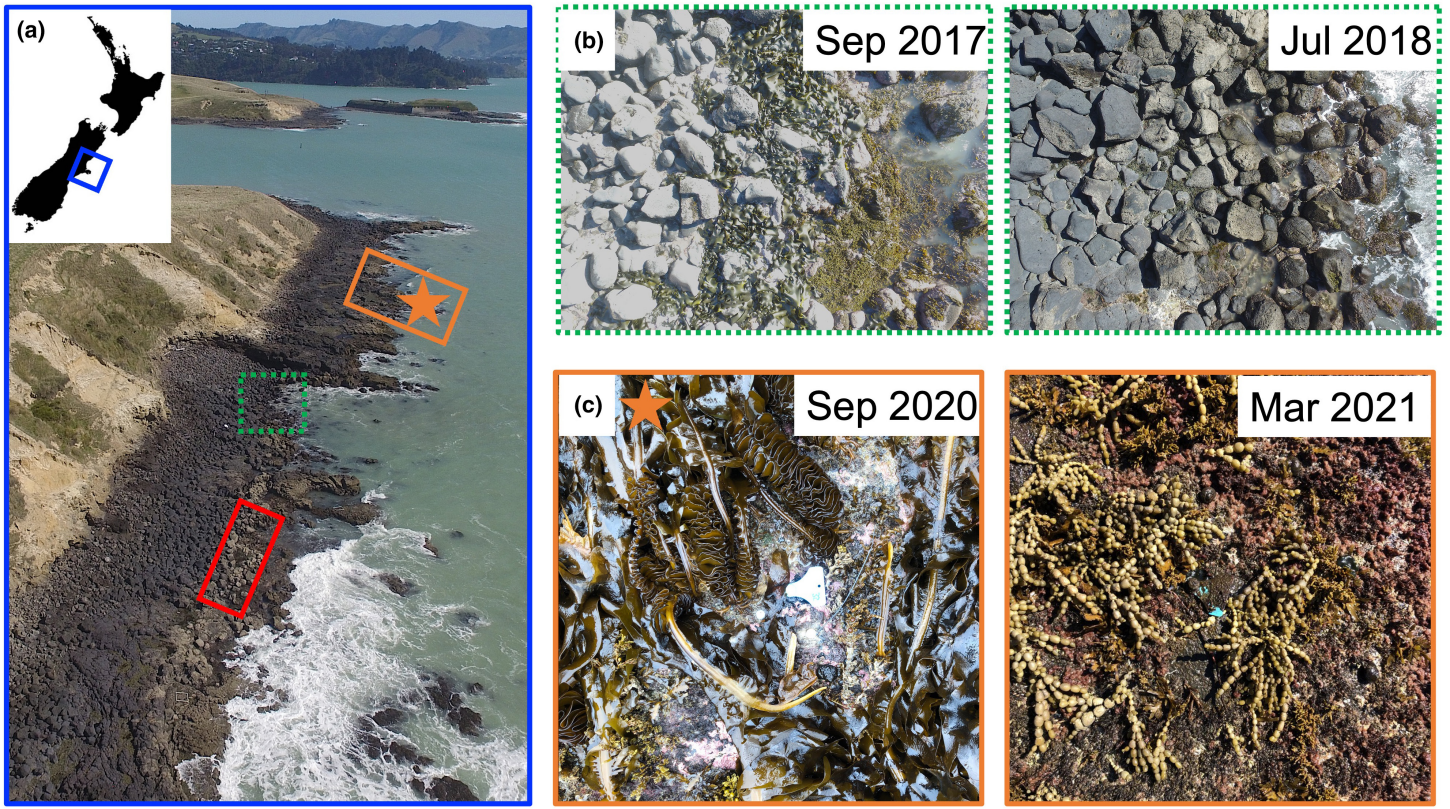
(a) Location of the rocky intertidal zone at Pile Bay (−43.615418, 172.765899) situated within Lyttelton Harbour, Banks Peninsula, New Zealand. Reef scale drone surveys were conducted along the entire high, mid and low intertidal zone. Site and patch scale surveys were done in 32 permanently marked plots from two reef sections where we had pre‐MHW photographs of understory species in 1 m^2^ plots. Reef section 1 is shown with a solid orange border (where 4 out of 16 plots were added *c*. 1 year later) and reef section 2 with a solid red border. The densest pre‐MHW *Durvillaea* sp. stands were found in the three marked squares. (b) Examples of two drone photographs taken before (2017) and after (2018) the MHW at a similar location based on geotagged coordinates (within the dotted green border in (a)) showing high cover of *Durvillaea* sp. before and 0% cover after the MHW. (c) Seasonal differences in algal cover at the same low intertidal permanent plot in spring (Sep 2020) and autumn (Mar 2021). Photographs show a clear change in dominant habitat‐forming fucoids and kelp. The orange star in (a) shows the exact location of the permanent plot at reef section 1.

Before the MHW in August 2017, six 1 m^2^ quadrats with 100% cover of bull kelp canopy were photographed at two bull kelp‐dominated sections of the reef (see Figure 1, i.e., a total of 12 quadrates). Before a photograph was taken, the large bull kelp fronds were pushed aside so that abundances of understory species could also be quantified. Within each photograph, percent cover of sessile organisms and counts of mobile organisms were recorded to their lowest taxonomic level (genus or species). Photographs were taken from random and scattered bull kelp plots covering the low‐to‐mid elevation zone on both reef sections, but plot‐specific elevation levels could not be determined from image analyses (and results are therefore only reported for the combined low‐mid elevation level). Furthermore, because pre‐MHW data were estimated from these photographs, some cryptic fauna like very small chitons, limpets, and snails, which can inhabit narrow cracks and crevices, may be underestimated compared with follow‐up post‐MHW in situ surveys (see below).

### 2.4 Impact of *Undaria*, elevation and season on succession (permanent plots)

To test how tidal elevation and invasive *Undaria* affected succession trajectories following the bull kelp extinction event, 0.25 m^2^ (0.5 × 0.5 m) permanent plots were established in habitats previously dominated by bull kelp on the two reef sections photographed in 2017. In March 2018, 12 permanent plots were marked on reef section 1 and 16 plots on section 2, with another 4 plots (that were not established initially due to bad weather, poor tides, and logistic problems) marked on reef section 2 in September 2020, to ensure the two reef sections had similar number of plots and sampling effort (Figure 1a). The elevation level of each plot above LAT was measured to nearest cm with an Abney level and reclassified to low (<0.7 m above LAT) or mid (>0.7 m above LAT) intertidal zones. In half of the permanent plots (eight per site), an *Undaria* removal treatment was applied where colonizing *Undaria* were regularly removed before sampling, and during sampling if recruits were found, to test whether this invasive species influenced postdisturbance succession trajectories. Originally, *Undaria* invaded all permanent plots and in 2018, when the *Undaria* removal treatment began, an average of 40 and 23 individuals were removed from plots in the low and mid zone, respectively. Within each permanent plot, percent cover of sessile organisms and counts of mobile organisms were recorded to their lowest taxonomic level (genus or species). Surveys were done during spring tides annually in spring (September) from 2018 to 2022 (1–5 year post‐MHW) to document long‐term changes and in autumn (March) from 2021 to 2023 (4–6‐year post‐MHW) to also test for seasonal variation.

Changes to community structure were visualized with nMDS, by combining data from the pre‐MHW photographs and the post‐MHW permanent plot surveys. Three‐factorial PERMANOVAs were done on the post‐MHW data only (because they were sampled differently than the pre‐MHW data) using annual spring data on multivariate community structure (using Bray–Curtis dissimilarity coefficients) and univariate responses (using Euclidian distances). Univariate responses included the abundances and taxonomic richness of all sessile (percent cover) and all mobile (counts) species, and key habitat‐forming species (percent cover), that is, *Undaria*, *Hormosira banksii*, *Cystophora torulosa*, *C. scalaris*, coralline turf, and encrusting coralline algae. Year (1‐, 2‐, 3‐, 4‐, and 5‐year post‐MHW) was considered a fixed factor and the factors *Undaria* removal (control and removal) and elevation (low and mid) were nested within a random plot factor. We supplemented the annual tests with repeated measures four‐way PERMANOVAs on the seasonal data (autumn and spring 2020–21 and 2022–23 using the same parameters as the three‐way PERMANOVA). However, the seasonal tests were less important for our hypothesis related to long‐term changes and were therefore only reported in online supplements. Again, data could not be transformed to variance homogeneity, but parametric PERMANOVA was (again) preferred over nonparametric ranked approaches for reasons detailed in Section 2.1. Significant test results were followed up with post hoc *t*‐test comparisons to identify significant treatments.

### 2.5 Habitat interactions (permanent plots)

To test whether habitat interactions in the post‐MHW community were affected by elevation and season “attachment surveys” were done in the 32 permanent plots, using a quadrat of 0.25 × 0.25 m (0.0625 m^2^) over 2 years from 2021 to 2023 (two times in spring and two times in autumn). For this survey, the small quadrat was placed in a random subsection of the larger 0.25 m^2^ permanent plot. “Attachments” were quantified as in Thomsen and South (2019) by recording all species combinations of flora and fauna (epibionts) attached to biogenic hosts, like epiphytic algae attached to seaweeds, or encrusting coralline algae growing on bivalves. Specifically, within each quadrat, all unique two‐species interactions were recorded (analogue to a richness metric) as well as the number of times each interaction occurred (analogue to an abundance metric). A three‐way PERMANOVA using Euclidian distances, on square‐root‐transformed data (to ensure variance homogeneity, *p* > .05 for all Levine test), was performed on the average number of attachments (“attachment abundances”) and their number of linkages (“attachment richness”) using the 2 years of seasonal data. The fixed factors year (2021–22 and 2022–23), season (autumn and spring), and elevation (low and mid) were nested in plots.

## 3 RESULTS

### 3.1 Changes to dominant seaweed on reef scale (drone images)

**FIGURE 2:**
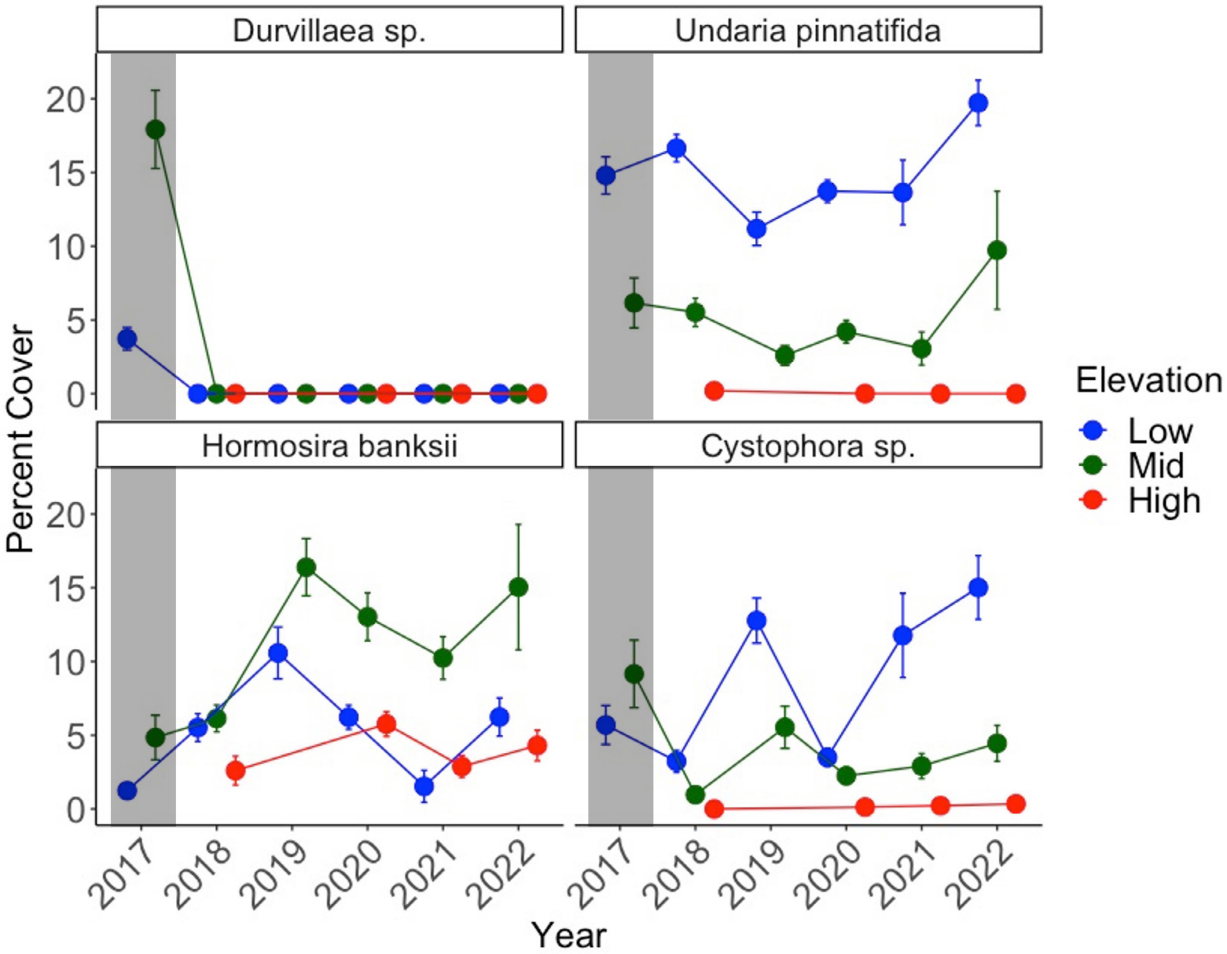
Average abundance (percent cover ± standard error) of four key habitat‐forming fucoids (*Cystophora* sp., including *C. torulosa* and *C. scalaris*, *Durvillaea* sp. and *H. banksii*) and kelp (*Undaria*) detected from reef scale drone imagery. Drone surveys were conducted annually between 2017 and 2022 during spring, when *Undaria* cover was highest. Average percent cover was calculated based on transects along the low (blue), mid (green), and high (red) intertidal zones. Data were not captured in the high intertidal zone in 2017 or 2019. The gray shading represents pre‐MHW coverages.

Before the 2017/18 MHW, the reef was dominated by habitat‐forming seaweeds, including *Durvillaea*, *Undaria*, *H banksii*, and *Cystophora* sp. (*C. torulosa* and *C. scalaris* could not be separated from drone images; Figure S1). *Durvillaea* and *Cystophora* sp. were significantly affected by the year × elevation interaction (*p*_*Durvillaea*_ = .001, 9% of Sums of Squares, *p*_*Cystophora* sp_ = 0.003, 3%, Table 1). In the case of *Durvillaea*, this is because it dominated the mid‐to‐low intertidal zone in 2017 (c. 18 ± 3% SE) before becoming locally extinct in 2018 (Table 1, Figure 2). Individual factors year and elevation also affected *Undaria* (*p*_Year_ = .001, 2%, *p*_Elevation_ = .001, 43%) and *H. banksii* (*p*_Elevation_ = .001, 12%). *Undaria* were dominant in the low intertidal zone both before (15 ± 1%) and immediately after (17 ± 1%) the MHW, whereas the mid zone became dominated by *H. banksii* (c. 6 ± 1%) and *Undaria* (6 ± 1%). Over the following 4 years (2019–2022), *Undaria* continued to dominate the low intertidal zone, reaching highest cover in 2022 (20 ± 1%) whereas *H. banksii* increased in cover in the mid zone to 17% (±2%) 3 years after the MHW and remained the dominant habitat‐former in 2022 (Table 1, Figure 2).

**TABLE 1:** Fixed two‐factorial permutational multivariate analysis of variance on percent cover of fucoid and kelp species analyzed by drone, using Bray–Curtis dissimilarity coefficients for multivariate data and Euclidian distance for univariate responses for (A) multivariate community structure, (B) *Durvillaea*, (C) *Undaria*, (D) *H. banksii* and (E) *Cystophora* sp.

	df	SS	% SS	*F*.model	*R* ^2^	*p*‐Value
A. Community						
Year	1	4.94	4.07	29.41	.04	**.001**
Elevation	2	32.95	27.13	98.00	.27	**.001**
Year × Elevation	2	2.01	1.66	5.98	.02	**.001**
Residuals	485	81.54	67.14		.67	
Total	490	121.45			1.00	
B. *Durvillaea*						
Year	1	1437.20	14.28	93.31	.14	**.001**
Elevation	2	273	2.71	8.86	.03	**.001**
Year × Elevation	2	881.30	8.76	28.61	.09	**.001**
Residuals	485	7470.70	74.25		.74	
Total	490	10,062.20			1.00	
C. *Undaria*						
Year	1	678	1.69	15.05	.02	**.001**
Elevation	2	17,458	43.60	193.65	.44	**.001**
Year × Elevation	2	38	0.09	0.43	.00	.657
Residuals	485	21,862	54.60		.55	
Total	490	40,037			1.00	
D. *H. banksii*						
Year	1	301	0.86	4.84	.01	.029
Elevation	2	4203	11.99	33.80	.12	**.001**
Year × Elevation	2	401	1.14	3.23	.01	.032
Residuals	485	30,151	86.01		.86	
Total	490	35,056			1.00	
E. *Cystophora* sp.						
Year	1	7	0.02	0.11	.00	.746
Elevation	2	3916	11.87	33.66	.12	**.001**
Year × Elevation	2	852	2.58	7.32	.03	**.003**
Residuals	485	28,209	85.53		.86	
Total	490	32,983			1.00	

*Note*: All factors were considered fixed, including year (2017, 2018, 2019, 2020, 2021, and 2022) and elevation (low, medium, and high). Significant *p*‐values are shown on bold (alpha < .01; variances were heterogenous).

### 3.2 Impact from *Undaria* and elevation on succession (understory photographs and permanent plots)

#### 3.2.1 Community

**FIGURE 3:**
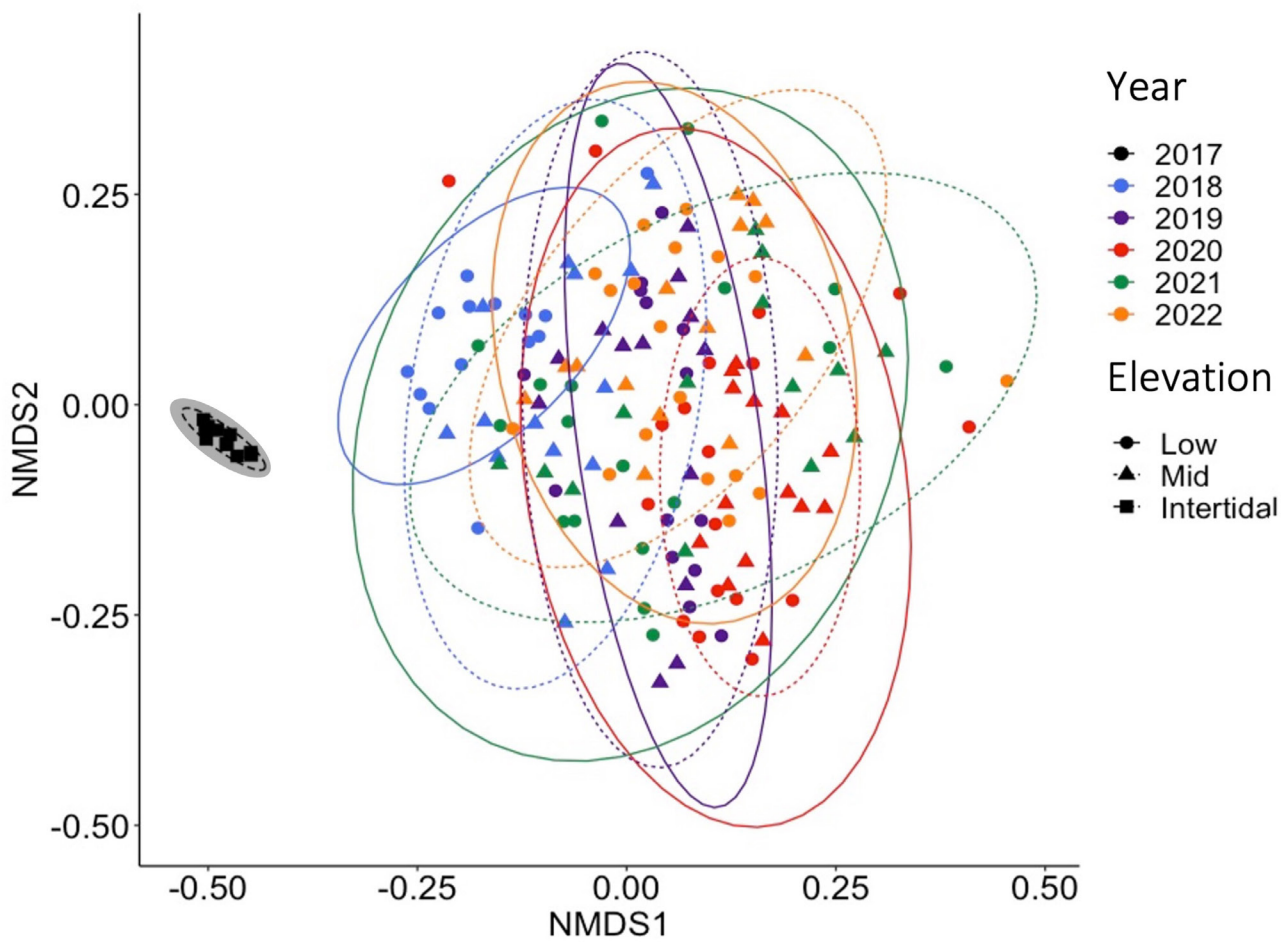
NMDS plot showing changes in community structure at sites previously dominated by bull kelp using pre‐MHW (2017) data from photographs of 1 m^2^ quadrats (*n* = 12) in the intertidal (black squares) and post‐MHW data from permanent 0.25 m^2^ plots (*n* = 32) separated into low (circles) and mid (triangles) intertidal elevations. The pre‐MHW data have a gray‐shaded background to highlight their extraction from photographs (where specific elevation levels could not be determined), whereas post‐MHW data were estimated from visual surveys of permanent plots and high‐precision elevation measurements.

**FIGURE 4:**
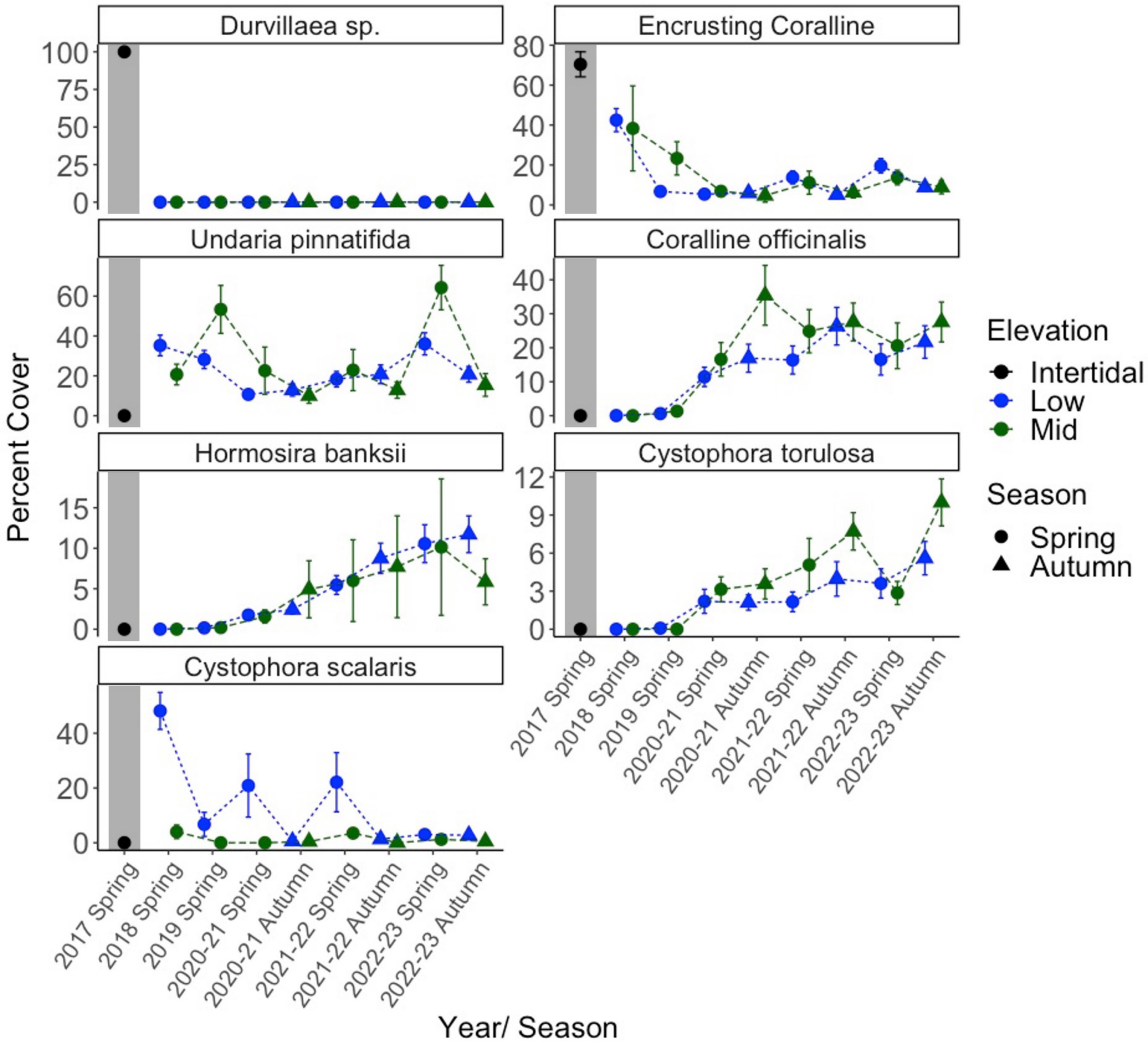
Average abundance (percent cover ± standard error) of key habitat‐forming fucoids (*Durvillaea* sp., *C. torulosa*, *C. scalaris*, and *H. banksii*) and kelp (*Undaria*), understory coralline turf (*C. officinalis*) and encrusting coralline algae, in photo quadrats before (2017, *n* = 12 1 m^2^ random plots, black points, gray shading, no elevation measurements) and after (*n* = 32 permanently marked 0.25 m^2^ plots, 2018–2022/23) the MHW, where post‐MHW data were separated into low (blue) and mid (green) intertidal elevations during spring (circles) and autumn (triangles). The abundance of *Undaria* only includes the data from the control plots where *Undaria* was not experimentally removed.

Community structures were very similar between the 2017 photographed quadrats, that is, before the MHW. However, in 2018, one year after the MHW, permanent plots had very different communities. Between 2018 and 2022, permanent plots remained distinct from the pre‐MHW community, and interplot distances increased, suggesting that more different species colonized into different elevation levels, creating more diverse communities at the reef scale (Figure 3). The PERMANOVA (on the post‐MHW community data only) showed significant individual effects of year (*p* = .001, 8% SS) and elevation (*p* = .001, 3%, Table 1). Taxa that explained most of the year effects included *C. officinalis* (18%–23%), encrusting coralline algae (19%–28%) and *H. banksii* (14%) whereas elevation effects were explained by *H. banksii* (11%–23%), *C. officinalis* (16%–22%), *Undaria* (12%), and encrusting coralline algae (17%). More specifically, composition of photographed quadrats before the MHW had 100% *Durvillaea* canopy cover and the understory was dominated by encrusting coralline algae (70 ± 6% SE; Figure 4), with a few bivalves (4 ± 2% SE) and serpulidae tube worms (1.5 ± 1% SE, Figure S2). There were also cnidarians (sea anemones, <0.1 ± <0.1%) and mobile mollusks, such as chitons, limpets, and snails (<0.1 ± <0.1% and 0.5 ± 0.25%, Figure S3). Composition in permanent plots, after the MHW, developed a mixed canopy of foundation species (see Section 3.2.3 and Figure 4). Furthermore, bivalves reached highest abundance in spring 2018 (6 ± 1% SE low elevation and 11 ± 3% SE mid elevation) but dropped to lowest levels in autumn 2022–23 (2 ± 0.5% SE low elevation and 1 ± 0.25% SE mid elevation). Serpulidae tube worms fluctuated in abundance, reaching highest levels in spring 2021–22 (4 ± 1% SE low elevation and 2 ± 1% SE mid elevation). Cnidarians and bryozoans remained low in abundance (Figure S2). Finally, mobile mollusks substantially increased after the MHW reaching highest levels in spring 2022–23 (28 ± 2 SE at low elevation and 20 ± 4 SE at mid elevation, Figure S3).

#### 3.2.2 Taxonomic richness and abundances of mobile and sessile species

**FIGURE 5:**
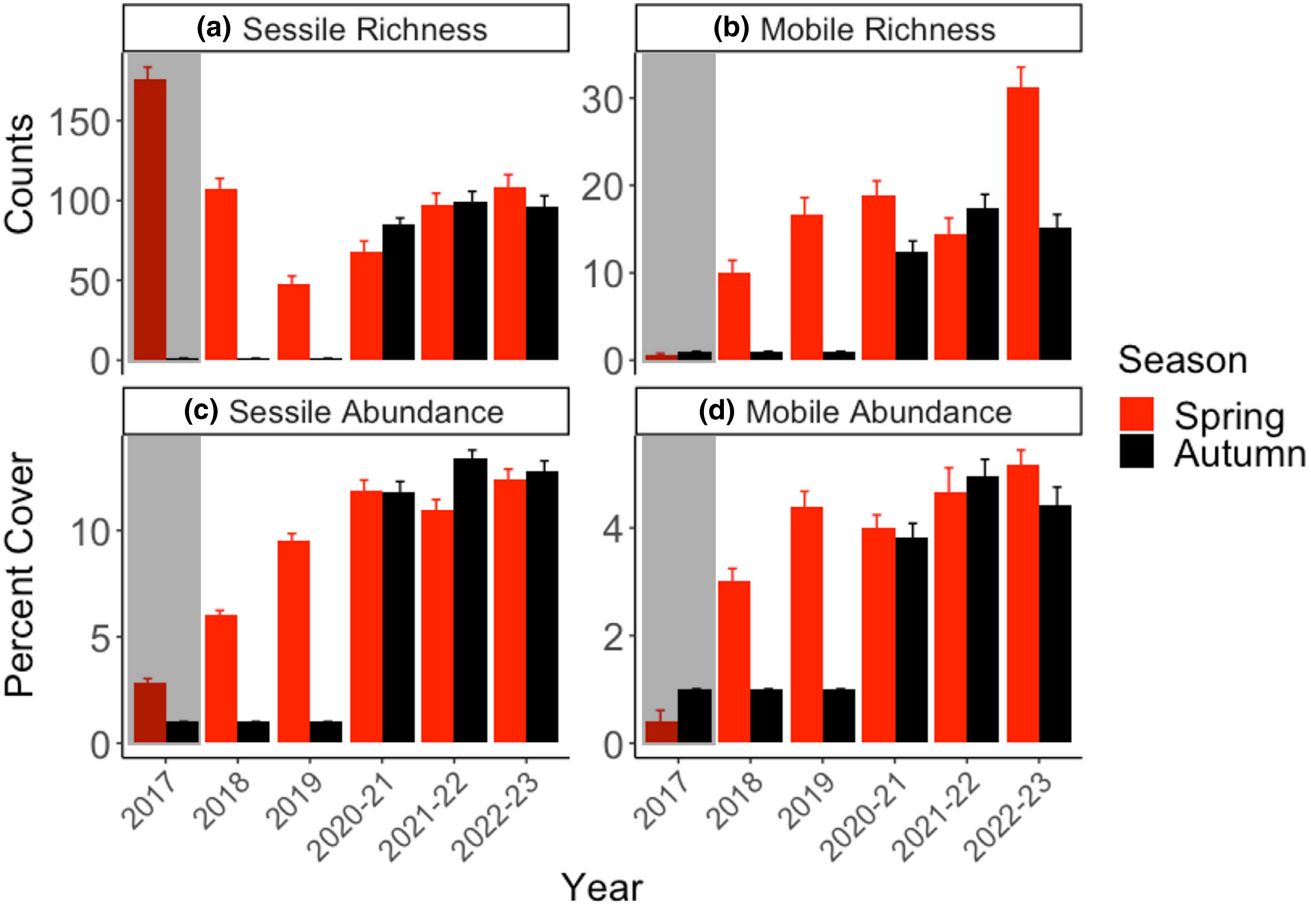
(a). Average sessile richness (counts ± standard error), (b) Average mobile taxonomic richness (counts ± standard error), (c) Average sessile abundance (percent cover ± standard error) of sessile organisms, and (d) Average mobile abundance (percent cover ± standard error), before (2017, *n* = 12 random 1 m^2^ plots, black points, gray shading, no elevation measurements) and after (*n* = 32 permanently marked 0.25 m^2^ plots, 2018–2022/23) the MHW during spring (red) and autumn (black).

The three main test factors explained in concert 69% (sessile richness), 56% (mobile richness), 72% (sessile abundance), and 68% (mobile abundance) of the total data variability. Overall, there was relatively high interplot variability (i.e., Plot (*Undaria* removal × elevation) tests were generally significant). We found no effect of elevation or *Undaria* removal (*p* > .01) and only a single significant interaction that explained little of data variability (Sessile abundance: *p*_year × elevation_ = .002, 5% of SS, Table 2D). By contrast, year was, as expected, significant across responses and explained a high proportion of the data variability (*p*_sessile richness_ = .001, 51% SS, *p*_mobile richness_ = .001, 17% SS, *p*_mobile abundance_ = .001, 35%, *p*_sessile abundance_ = .001, 28%, Table 2A–D). More specifically, sessile (Figure 5a) and mobile (Figure 5b) richness increased from low pre‐MHW (3 ± 0.2 and 0.4 ± 0.2 taxa, respectively) to highest levels in 2022 (12 ± 0.5 and 5 ± 0.3 taxa). Post hoc comparisons showed significant contrasts between most years (excluding 2020 vs. 2021 and 2020 vs. 2022 for sessile richness and excluding 2019 vs. 2020, 2019 vs. 2021, 2020 vs. 2021, and 2021 vs. 2022 for mobile richness). By comparison, sessile abundance was highest in 2017 (175 ± 8%, including 100% cover of bull kelp canopies) but declined after the MHW in 2018 (107 ± 7%) and 2019 (47 ± 5%), increased in 2020, and reached its highest levels post‐MHW in 2022 (>108 ± 7%) (Figure 5c). Finally, mobile fauna abundances (Figure 5d) increased from lowest levels pre‐MHW in 2017 (0.5 ± 0.2 individuals) to highest levels in 2022 (31 ± 2 individuals). Post hoc tests showed again that most years were different for both sessile (minus 2018 vs. 2022 and 2021 vs. 2022) and mobile species abundance (excluding 2019 vs. 2020).

**TABLE 2:** Repeated measures three‐factorial permutational (999 permutations) multivariate analysis of variance on multivariate (A) community structure using Bray Curtis similarity and univariate sessile and mobile richness and abundance using Euclidian Distance for (B) sessile richness, (C) mobile richness, (D) sessile abundance and (E) mobile abundance, within permanent plots surveyed annually in spring after the MHW, between 2018 and 2022.

	df	SS	% SS	*F*.model	*R* ^2^	*p*‐Value
A. Community						
*Undaria* removal	1	0.31	1.01	1.71	.01	.089
Elevation	1	0.84	2.73	4.59	.03	**.001**
Year	1	2.39	7.76	13.05	.08	**.001**
Elevation × *Undaria* removal	1	0.24	0.78	1.32	.01	.208
Year × *Undaria* removal	1	0.27	0.88	1.48	.01	.146
Year × Elevation	1	0.22	0.72	1.21	.01	.258
Year × Elevation × *Undaria* removal	1	0.16	0.51	0.85	.01	.556
Residuals	144	26.36	85.61	0.86		
Total	151	30.79				

	df	SS	% SS	Pseudo‐*F*	*p*‐Value
B. Sessile richness					
*Undaria* removal	1	1.69	0.10	0.15	.713
Elevation	1	0.51	0.03	0.04	.829
Year	4	842.30	51.05	42.57	**.001**
Elevation × *Undaria* removal	1	1.24	0.08	0.11	.757
Year × *Undaria* removal	4	8.47	0.51	0.43	.792
Year × Elevation	4	31.59	1.91	1.60	.187
Plot (*Undaria* removal × Elevation)	28	327.30	19.84	2.36	**.001**
Year × Elevation × *Undaria* removal	4	8.34	0.51	0.42	.786
Plot × Year × Elevation × *Undaria* removal	104	514.48	31.18	No test	No test
C. Mobile richness					
*Undaria* removal	1	0	0	0	.988
Elevation	1	6.68	1.27	1.14	.295
Year	4	89.70	17.10	10.19	**.001**
Elevation × *Undaria* removal	1	2.01	0.38	0.34	.595
Year × *Undaria* removal	4	12.94	2.47	1.47	.227
Year × Elevation	4	7.13	1.36	0.81	.527
Plot (*Undaria* removal × Elevation)	28	166.63	31.78	2.70	**.002**
Year × Elevation × *Undaria* removal	4	19.99	3.81	2.27	.071
Plot × Year × Elevation × *Undaria* removal	104	228.96	43.66	No test	No test
D. Sessile abundance					
*Undaria* removal	1	238.21	0.08	0.07	.791
Elevation	1	6475.60	2.27	1.80	.193
Year	4	80,971	28.33	26.08	**.001**
Elevation × *Undaria* removal	1	1687.60	0.59	0.47	.502
Year × *Undaria* removal	4	2351.20	0.82	0.76	.567
Year × Elevation	4	13,827	4.84	4.45	**.002**
Plot (*Undaria* removal × Elevation)	28	102,330	35.80	4.71	**.001**
Year × Elevation × *Undaria* removal	4	1757.90	0.62	0.57	.673
Plot × Year × Elevation × *Undaria* removal	104	80,709	28.24	No test	No test
E. Mobile abundance					
*Undaria* removal	1	61.03	0.26	0.27	.598
Elevation	1	442.28	1.89	1.97	.209
Year	4	8088.80	34.61	28.25	**.001**
Elevation × *Undaria* removal	1	238.45	1.02	1.06	.345
Year × *Undaria* removal	4	382.89	1.64	1.34	.263
Year × Elevation	4	474.85	2.03	1.66	.163
Plot (*Undaria* removal × Elevation)	28	6392.10	27.35	3.19	**.001**
Year × Elevation × *Undaria* removal	4	155.34	0.66	0.54	.738
Plot × Year × Elevation × *Undaria* removal	104	7445	31.86	No test	No test

*Note*: Year (2018, 2019, 2020, 2021, and 2022), *Undaria* removal (control and removal), and elevation (low and mid) were all fixed factors, where the latter two were nested within the random repeated measures plot factor. Significant *p*‐values are shown on bold (alpha < .01; variances were heterogenous).

#### 3.2.3 Abundances of key species

After the MHW, percent cover in the permanent plots was dominated by smaller fucoids (*C. torulosa*, *C. scalaris*, and *H. banksii*), *Undaria*, and understory *C. officinalis* and encrusting coralline algae. The three test factors explained in concert 61% (encrusting coralline), 64% (*Undaria*), 58% (*C. officinalis*), 65% (*H. banksii*), 59% (*C. torulosa*), and 62% (*C. scalaris*) of the total data variability (Table 3). Again, we found high interplot variability across tests (all Plot (*Undaria* removal × elevation) tests were significant) but only few significant interactions that explained little of the data variability (*Undaria*: year × *Undaria* removal *p* = .009, 5%, year × elevation *p* = .002, 6%). Furthermore, only *Undaria* was affected by elevation, being most abundant in the low zone (*p* = .001, 9%). By contrast, all species were strongly affected by year (*p*_encrusting coralline_ = .001, 35%, *p*_*Undaria*_ = .001, 9%, *p*_*H. banksii*_ = .001, 21%, *p*_*C. torulosa*_ = .001, 20%, *p*_*C. officinalis*_ = .001, 18%, *p*_*C. scalaris*_ = .001, 12%) (Table 3). Specifically, encrusting coralline algae decreased from high pre‐MHW (70 ± 6%) to lowest levels in 2019 (23 ± 8% and 7 ± 2% in the mid and low zone, respectively). Following the loss of bull kelp and encrusting coralline algae, *Undaria* became the dominant foundation species in the low zone, increasing to highest levels in 2018 (48 ± 7% and 4 ± 2% in the low and mid zone, respectively). After the MHW, *C. officinalis* became the dominant understory species, reaching highest levels in 2022 (64 ± 6% and 36 ± 6% in the mid and low zone, respectively). A few years post‐MHW, smaller native fucoids colonized the pre‐MHW bull kelp‐dominated plots, where *H. banksii* became the dominant mid zone foundation species increasing to highest levels in autumn 2020 (35 ± 9% and 17 ± 4% in the mid and low zone, respectively). *C. scalaris* increased to highest levels in autumn 2021 (7 ± 1% and 4 ± 1% in the mid and low zone, respectively). Finally, *C. torulosa* increased to highest levels in spring 2022 (11 ± 2% and 10 ± 8% in the low and mid zone, respectively; Figure 4). Post hoc comparisons showed significant contrasts between all years for *C. torulosa* and all years excluding 2021 vs. 2022 for *H. banksii*. Other species showed significant contrasts for most years excluding 2019 vs. 2020 and 2021 vs. 2022 for encrusting coralline algae and 2018 vs. 2019, 2018 vs. 2022 and 2020 vs. 2021 for *C. officinalis*. Finally, *C. scalaris* and *Undaria* showed significant contrasts between years only 60% and 40% of the time, respectively.

**TABLE 3:** Repeated measures three‐factorial permutational (999 permutations) multivariate analysis of variance on percent cover of five key habitat‐forming seaweed species within permanent plots surveyed annually in spring after the MHW, between 2018 and 2022, using Euclidian distance for univariate responses (A) *H. banksii*, (B) *Undaria*, (C) *C. torulosa*, (D) *C. scalaris*, (E) *C. officinalis* and (F) encrusting coralline.

	df	SS	% SS	Pseudo‐*F*	*p*‐Value
A. *H. banksii*					
*Undaria* removal	1	360.27	0.85	0.68	.412
Elevation	1	882.38	2.08	1.67	.228
Year	4	9071	21.41	16.04	**.001**
Elevation × *Undaria* removal	1	1.45	0	0	.954
Year × *Undaria* removal	4	350.48	0.83	0.62	.671
Year × Elevation	4	900.50	2.13	1.59	.179
Plot (*Undaria* removal × Elevation)	28	15,046	35.51	3.80	**.001**
Year × Elevation × *Undaria* removal	4	630.51	1.49	1.11	.361
Plot × Year × Elevation × *Undaria* removal	104	14,705	34.71	No test	No test
B. *Undaria*					
*Undaria* removal	1	1521.2	2.75	2.75	.110
Elevation	1	7704.2	13.91	13.90	**.001**
Year	4	5096	9.20	6.56	**.001**
Elevation × *Undaria* removal	1	1728	3.12	3.12	.090
Year × *Undaria* removal	4	2604.2	4.70	3.35	**.009**
Year × Elevation	4	3345.9	6.04	4.31	**.002**
Plot (*Undaria* removal × Elevation)	28	15,733	28.40	2.90	**.001**
Year × Elevation × *Undaria* removal	4	1688.5	3.05	2.18	.078
Plot × Year × Elevation × *Undaria* removal	104	20,183	36.44	No test	No test
C. *C. torulosa*					
*Undaria* removal	1	40.29	0.38	0.31	.629
Elevation	1	85.01	0.79	0.65	.477
Year	4	2140.2	19.94	12.73	**.001**
Elevation × *Undaria* removal	1	47.12	0.44	0.36	.606
Year × *Undaria* removal	4	36.80	0.34	0.22	.941
Year × Elevation	4	79.52	0.74	0.47	.768
Plot (*Undaria* removal × Elevation)	28	3738.3	34.82	3.18	**.001**
Year × Elevation × *Undaria* removal	4	29.97	0.28	0.18	.961
Plot × Year × Elevation × *Undaria* removal	104	4372.8	40.73	No test	No test
D. *C. scalaris*					
*Undaria* removal	1	3.13	0.14	0.10	.784
Elevation	1	13.11	0.57	0.42	.582
Year	4	288.44	12.44	8.59	**.001**
Elevation × *Undaria* removal	1	120.48	5.19	3.85	.040
Year × *Undaria* removal	4	9.49	0.41	0.28	.904
Year × Elevation	4	24.63	1.06	0.73	.584
Plot (*Undaria* removal × Elevation)	28	890.82	38.41	3.79	**.001**
Year × Elevation × *Undaria* removal	4	65.52	2.82	1.95	.106
Plot × Year × Elevation × *Undaria* removal	104	873.08	37.64	No test	No test
E. *C. officinalis*					
*Undaria* removal	1	143.28	0.14	0.11	.742
Elevation	1	18.67	0.02	0.01	.914
Year	4	18,323	18.41	11.56	**.001**
Elevation × *Undaria* removal	1	117.8	0.12	0.09	.781
Year × *Undaria* removal	4	1469.5	1.48	0.93	.475
Year × Elevation	4	888.52	0.89	0.56	.670
Plot (*Undaria* removal × Elevation)	28	35,624	35.8	3.21	**.001**
Year × Elevation × *Undaria* removal	4	1280.7	1.29	0.81	.564
Plot × Year × Elevation × *Undaria* removal	104	41,211	41.41	No test	No test
F. Encrusting Coralline					
*Undaria* removal	1	4.56	0.01	0.01	.913
Elevation	1	227.08	0.34	0.55	.491
Year	4	23,111	34.47	23.13	**.001**
Elevation × *Undaria* removal	1	341.61	0.51	0.83	.382
Year × *Undaria* removal	4	746.51	1.11	0.75	.556
Year × Elevation	4	1699.3	2.53	1.70	.146
Plot (*Undaria* removal × Elevation)	28	11,610	17.32	1.66	.039
Year × Elevation × *Undaria* removal	4	1875.4	2.80	1.88	.123
Plot × Year × Elevation × *Undaria* removal	104	25,983	38.75	No test	No test

*Note*: The year factor (2018, 2019, 2020, 2021, and 2022) was fixed and factors *Undaria* removal (control and removal), and elevation (low and mid) were nested within the random factor plot (a repeated measure). Significant *p*‐values are shown on bold (alpha < .01; variances were heterogenous).

### 3.3 Habitat interactions (permanent plots)

**FIGURE 6:**
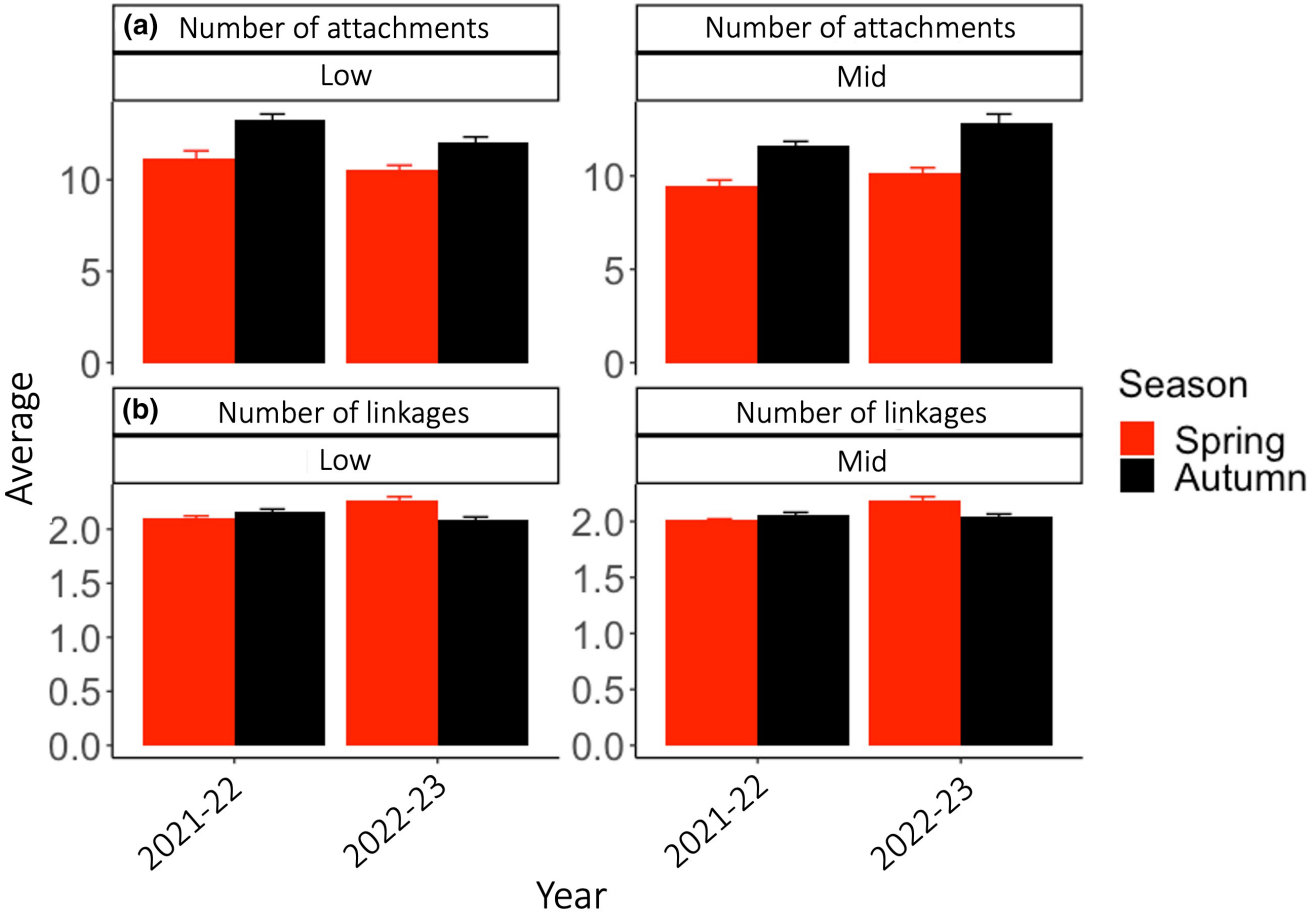
(a) Average number of habitat attachments (± standard error, proxy for abundance) and (b) Average number of linkages (± standard error, proxy for richness of species involved in habitat interactions) within permanent plots during seasonal surveys in spring (red) and autumn (black) between 2021–22 and 2022–23.

We found more attachment interactions in the low compared to mid zone and in autumn compared to spring (Figure 6a), whereas the analyses on the number of linkages revealed more complicated results (Figure 6b). The three test factors explained in concert 7% and 5% of the data variability, for number of attachment and linkages, respectively (Table 4). We found significant interactions for year × elevation for attachments (*p* = .011, 1% of SS) and year × season for linkages (*p* = .001, 2%). Furthermore, year was only significant for linkages (*p* = .006, 0.6%) whereas linkages and attachments were both affected by season (*p*_linkages_ = .001, 0.7%, *p*_attachments_ = .001, 5%) and elevation (*p*_attachments_ = .001, 0.6%, *p*_linkages_ = .001, 1%) (Table 4). Most attachment chains involved only two individuals, but a few had three or more species, for example, the three‐chain interaction with *H. banksii*‐attached‐to‐*C. maschalocarpum‐*attached‐to‐*C. officinalis* was observed 165 times (pooled across years, seasons, and elevations). The second most common interaction was the *C. torulosa‐*attached‐to*‐C. officinalis* chain (62 times), followed by the snail *Lunella smaragda‐*attached‐to‐*H. banksii* (54 times), *Lunella smaragda‐*attached‐to‐*C. officinalis* (53 times), and *Colpomenia‐*attached‐to‐*C. officinalis* (44 times). The longest chain involved five species and was found only once: *C. scalaris‐*attached‐to‐*C. maschalocarpum*‐attached‐to‐*Aulacomya maoriana*‐attached‐to*‐C. officinalis*‐attached‐to‐encrusting coralline alga.

**TABLE 4:** Three‐factor analysis of variance on square‐root‐transformed average number of habitat attachments and number of linkages within attachments, surveyed seasonally in autumn and spring after the MHW for (A) number of attachments, and (B) number of linkages.

	df	SS	*F*‐value	*p*‐Value
A. Number of attachments				
Year	1	0.000	0.025	.874
Season	1	2.150	73.260	**<.001**
Elevation	1	0.160	5.529	**.019**
Year × Season	1	0.040	1.200	.274
Year × Elevation	1	0.190	6.502	**.011**
Season × Elevation	1	0.040	1.311	.253
Year × Season × Elevation	1	0.030	0.887	.347
Residuals	1236	36.240		
B. Number of linkages				
Year	1	0.013	7.349	**.007**
Season	1	0.017	9.487	**.002**
Elevation	1	0.023	13.147	**<.001**
Year × Season	1	0.050	28.995	**<.001**
Year × Elevation	1	0.001	0.615	.433
Season × Elevation	1	0.000	0.223	.637
Year × Season × Elevation	1	0.001	0.334	.564
Residuals	1236	2.144		

*Note*: The factors year (2021–22 and 2022–23), season (autumn and spring) and elevation (low and mid) were all fixed. Significant *p*‐values are shown on bold (alpha < .05).

## 4 DISCUSSION

The rise in extreme MHWs across the globe, and their ecological impacts, is of growing concern, especially when these events cause the loss of habitat‐forming foundation species (Arafeh‐Dalmau et al., 2019; Straub et al., 2019; Tait et al., 2021; Thomsen et al., 2019; Wernberg, 2021; Wernberg et al., 2016). Here, we investigated community shifts after an extreme MHW resulted in the local extinction of an important foundation species. The community was surveyed over 6 years and impacts associated with annual, seasonal, and elevational test factors were quantified. We found that the extreme MHW caused long‐lasting, possibly permanent, community changes to a coastal ecosystem after the dominant foundation species was lost, triggering a shift in understory species, and followed by high recruitment and replacement by alternative foundation species, that, in concert, likely will inhibit future recolonization of lost species (South et al., 2017; Thompson & Schiel, 2012; Thomsen et al., 2021; Wernberg, 2021). Until recently, little was known about long‐term ecological effects driven by MHWs, although slow and relatively minor reorganization of community structures has been observed where the dominant foundation species survive and at least partly recover (Spiecker & Menge, 2022; Suryan et al., 2021; Whalen et al., 2023). The shortage of long‐term impact studies is likely because the most extreme MHWs have only occurred over the last 15 years (Sen Gupta et al., 2020), so MHW‐associated loss of habitat‐forming species has only been reported within the last decade (Arafeh‐Dalmau et al., 2019; Filbee‐Dexter et al., 2020; Rogers‐Bennett & Catton, 2019; Smale et al., 2019; Smale & Wernberg, 2013; Smith et al., 2023; Straub et al., 2019; Tait et al., 2021; Thomsen et al., 2019; Wernberg et al., 2013, 2016), even though loss of seaweed habitats (to other stressors) is not a new phenomenon (e.g., Gunnill, 1985; Laurie, 1990; Murray & Horn, 1989; Soto‐Mamani, 1985). Importantly, to our knowledge no studies have documented long‐term succession trajectories after extirpation of dominant foundation species following extreme MHWs, or shown shifting dominance between newly colonized invasive and native (alternative) foundation species, until now.

Globally, research suggests that localized extinctions can result in slow recovery of the once‐dominant canopy‐former (Straub et al., 2019). However, here we found support for our hypothesis that bull kelp did not return to Pile Bay reefs during a sampling period of 6 years, and bull kelp recruits are yet to be found in this region (pers. obs. May 2023, the nearest surviving population remain >10 km away). While bull kelp has effective settlement of fertilized eggs over short distances (meters) and short time scales (hours), hardly any zygotes disperse longer distances (Fraser et al., 2018; Taylor & Schiel, 2003; Velásquez et al., 2020). Therefore, reef‐to‐reef recolonization is more likely to arise from detached fertile male and female thalli arriving as intertidal beach cast at the exact same time and place (Velásquez et al., 2020). Furthermore, colonization by coralline turf, *Undaria* and smaller canopy‐formers, can inhibit bull kelp recruitment because zygotes and juveniles settle and survive best on bare rock and encrusting alga (Schiel, 2019; Taylor & Schiel, 2005). In short, it is not surprising that bull kelp have not recolonized Pile Bay, even though this location is positioned well within *D. poha* latitudinal range (its northern range limit is 200 km north; Fraser et al., 2018, 2012; Taylor & Schiel, 2003; Velásquez et al., 2020). Similar severe and long‐lasting impacts from a MHW have been documented in Western Australia, where lost foundation species have not recolonized 8 years after an extreme event caused c. 100 km range contractions of the kelp *Ecklonia radiata* and the fucoid *Scytothalia dorycarpa* (Smale & Wernberg, 2013; Straub et al., 2019; Wernberg, 2021; Wernberg et al., 2016).

In our study, bull kelp was replaced by different seaweed and animals at slightly different elevations, which resulted in higher net biodiversity and abundance of mobile fauna and sessile understory seaweeds (minus those associated with bull kelp like encrusting coralline algae). Some of these species are “alternative foundation species,” which, like bull kelp, provide habitat for plants and animals and alter abiotic conditions, for example, by reducing light and increasing relative humidity in the subcanopy (Elsberry & Bracken, 2021; Lilley & Schiel, 2006; Thomsen et al., 2019). However, in contrast to heavy and large‐bladed bull kelp, canopies of the alternative foundation species do not “whiplash” the substrate preventing some individuals from inhabiting the substrate underneath (Stevens et al., 2002; Taylor & Schiel, 2010), partly because they are much smaller (0.5–2 m). Indeed, bull kelps are the largest fucoid seaweed worldwide, with fronds up to 10 m in length and biomass reaching 80 kg m^−2^ (Santelices et al., 1980; Taylor & Schiel, 2005), and the fronds create a whiplash effect under wavy conditions that maintain a unique understory of encrusting alga, certain epiphytes and mollusk, like abalone and chitons (Santelices et al., 1980; Schiel, 2019; Stevens et al., 2002; Taylor & Schiel, 2010; Thomsen & South, 2019). Furthermore, although the alternative foundation species benefitted after bull kelp was lost, endemic *D. poha* has a narrow latitudinal range (Fraser et al., 2012) and appears to be vulnerable to extreme MHWs (Thomsen et al., 2019, 2021), which will have cascading effects on diversity and abundance of the many culturally and economically important species they support, like fish and abalone, as well as the multitude of grazing invertebrates that reside in their large holdfasts (Fraser et al., 2011; Hay, 1977; Santelices, 1990; Schiel et al., 2018; Taylor & Schiel, 2005).

Intertidal habitats vary dramatically in environmental conditions across short vertical gradients, as desiccation and temperature fluctuations increase with shore height, but competition for space and predation by marine consumers decreases (Davenport & Davenport, 2005; Raffaelli & Hawkins, 1996; Schreider et al., 2003; Thomsen et al., 2016). However, our analysis showed relatively minor effects of elevation on new colonization by alternative foundation species, although a more diverse benthic understory community developed 3–4 years after the extinction event, with vertical (niche) partitioning as different subordinate species colonised slightly different elevation levels (that previously was dominated by bull kelp). For example, coralline turf and *Undaria* immediately colonized and dominated in the mid‐intertidal zone, whereas interspersed *H. banksii* and *Cystophora* sp. became the dominant habitat‐formers in the mid‐to‐high intertidal zone, as found in past clearance experiments (Lilley & Schiel, 2006; Schiel & Lilley, 2011). We also found, as hypothesized, that *Undaria* continued to benefit from open spaces across elevations until 2019, whereafter bare space decreased (as more species colonized), and *Undaria* became less common in higher tidal elevations—eventually matching its typical vertical distribution near the low water neap tide mark (Forrest & Taylor, 2002; South et al., 2015). Indeed, *Undaria* was the only alternative foundation species that was significantly affected by elevation. To our knowledge, mechanisms driving *Undaria's* upper vertical limit have not yet been experimentally investigated but are likely caused by desiccation stress and perhaps also competition with other intertidal algae and invertebrate grazing (South et al., 2017).

We also showed that season significantly affected sessile taxonomic richness and mobile species abundance, as well as the abundance of four key habitat‐forming species and understory corallines (see Appendix S1), probably because of cyclic changes in external environmental conditions like dissolved nutrient concentration, temperature, light availability, and wave action (Dayton, 1985; Lilley & Schiel, 2006; Schiel & Lilley, 2011). Indeed, large perennial brown alga, like kelp and fucoids, have complex life histories evolved to respond to these predicable changes (Clayton, 1988; Schiel & Foster, 2006). For example, invasive *Undaria* is a winter annual species with a sporophyte that grows rapidly during winter and spring forming relatively short‐lived but dense monospecific stands (South et al., 2017). By contrast, native *H. banksii* and *Cystophora* sp. colonized more slowly, but their perennial canopies provide temporally more stable biogenic habitats for epiphytes and mobile fauna, and can therefore increase the diversity and stability of attachment networks (Thomsen & South, 2019). We also found that *Undaria* became less successful in colonizing the mid‐intertidal zone over time, for example, an average of 40 and 23 individuals were removed from the *Undaria* removal plots in the low and mid zone plots in 2018, compared with only 11 and 0 in spring 2022. This lowered recruitment success supports past studies that also conclude that *Undaria* is most efficient in colonizing disturbed open space habitats and can be suppressed by native canopies (which, in our study, developed over time; Epstein & Smale, 2017; Schiel, 2019; Schiel et al., 2018; South et al., 2015, 2017; South & Thomsen, 2016; Taylor & Schiel, 2005; Thompson & Schiel, 2012; Thomsen & South, 2019). These same experimental studies also supported our findings that *Undaria* have relatively little impact on native species successional trajectories, probably because of the species pronounced seasonality, that allowed native species to colonize plots in spring and summer (when *Undaria* is sparse or absent). Finally, we showed that species‐attachment interactions were more common in the low zone during autumn when plots have lower *Undaria* cover but higher cover of mixed *H. banksii* and *Cystophora* sp. canopies, and understory coralline turf, where the latter two taxa provide habitat for many epiphytes (like the brooding anemone *Cricophorus nutrix*) and mobile snails and whelks, like in Thomsen and South (2019). Unfortunately, we did not have habitat‐interaction data from the pre‐MHW bull kelp bed, but other research suggests that bull kelp typically facilitate encrusting alga (by whiplashing more competitively dominant seaweed), which subsequently provide habitat for a few red seaweed like *Ballia* spp., *Gelidium microphyllum* and various limpets and chitons through facilitation cascades (Schiel, 2019; Taylor & Schiel, 2005; Thomsen & South, 2019). These specific bull kelp community interactions have largely been lost after the MHW and are instead replaced with different interaction networks now driven by *Cystophora* sp. and coralline turf (Thomsen & South, 2019). Furthermore, these types of attachment interactions highlight the importance of facilitation cascades in modifying and enhancing marine biodiversity (Altieri et al., 2007; Gribben et al., 2019; Thomsen et al., 2010).

In conclusion, we documented long‐term successional community changes following the extirpation of a temperate marine foundation species, driven by an extreme MHW. Specifically, lost endemic and native bull kelp did not recover over 6 years but was instead replaced by invasive and native alternative foundation species that altered interaction networks and increased diversity of understory flora and fauna, like coralline turf, that can inhibit future recolonization of bull kelp. As MHWs are predicted to become longer and stronger, localized extinction events and similar conspicuous changes to communities and associated ecosystem functions and services (Smith et al., 2021) are expected to become increasingly common and will be a major threat to endemic cold‐water species with narrow latitudinal ranges, like *D. poha*.

## AUTHOR CONTRIBUTIONS

**Shinae Montie:** Conceptualization (equal); formal analysis (lead); investigation (lead); methodology (lead); visualization (equal); writing – original draft (lead); writing – review and editing (equal). **Mads S. Thomsen:** Conceptualization (equal); funding acquisition (lead); supervision (lead); visualization (equal); writing – review and editing (equal).

## CONFLICT OF INTEREST STATEMENT

The authors declare that the research was conducted in the absence of any commercial or financial relationships that could be construed as a potential conflict of interest.

## Supporting information


Appendix S1

## Data Availability

The data that support the findings of this study are openly available in “figshare” at https://doi.org/10.6084/m9.figshare.23519679.v1.
