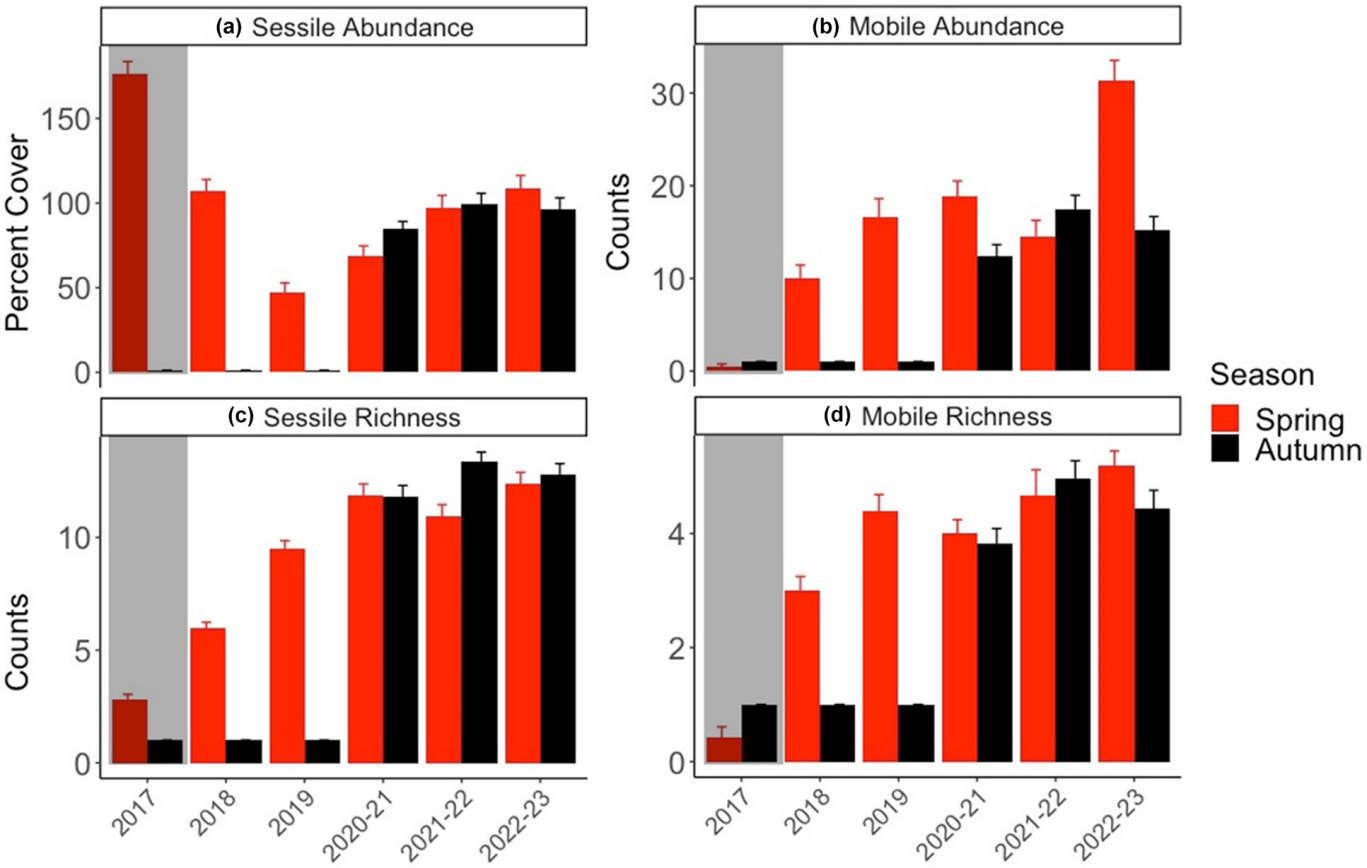# Correction to “Long‐term community shifts driven by local extinction of an iconic foundation species following an extreme marine heatwave”

**DOI:** 10.1002/ece3.10584

**Published:** 2023-10-20

Montie, S., & Thomsen, M. S. (2023). Long‐term community shifts driven by local extinction of an iconic foundation species following an extreme marine heatwave. Ecology and Evolution, 13(6), e10235.

In Figure 5, the labels are incorrect. Figure 5a and 5b shows average abundance of sessile and mobile organisms, respectively. Figure 5c and 5d shows average taxonomic richness of sessile and mobile organisms, respectively.

The figure caption should read “(a) Average abundance (percent cover ± standard error) of sessile organisms, (b) average abundance (counts ± standard error) of mobile organisms, (c) average sessile taxonomic richness (counts ± standard error), and (d) average mobile taxonomic richness (counts ± standard error), before (2017, n = 12 random 1 m2 plots, black points, gray shading, no elevation measurements) and after (n = 32 permanently marked 0.25 m2 plots, 2018–2022/23) the MHW during spring (red) and autumn (black).

We apologize for this error.