# Supplementary material for: Long‐term community shifts driven by local extinction of an iconic foundation species following an extreme marine heatwave
Source: Ecol Evol. 2023 Jun 26;13(6):e10235. doi: 10.1002/ece3.10235 (PMC10293786; doi:10.1002/ece3.10235)
Supplement: Supplementary file 1 — Appendix S1 [file ECE3-13-e10235-s001.docx]

**Supplement Material**

# **Figures**

**Figure S1.** Average abundance (percent cover ± standard error) for all drone detected seaweed species and groups of species pooled across elevation for the entire reef at Pile Bay before the MHW in 2017.


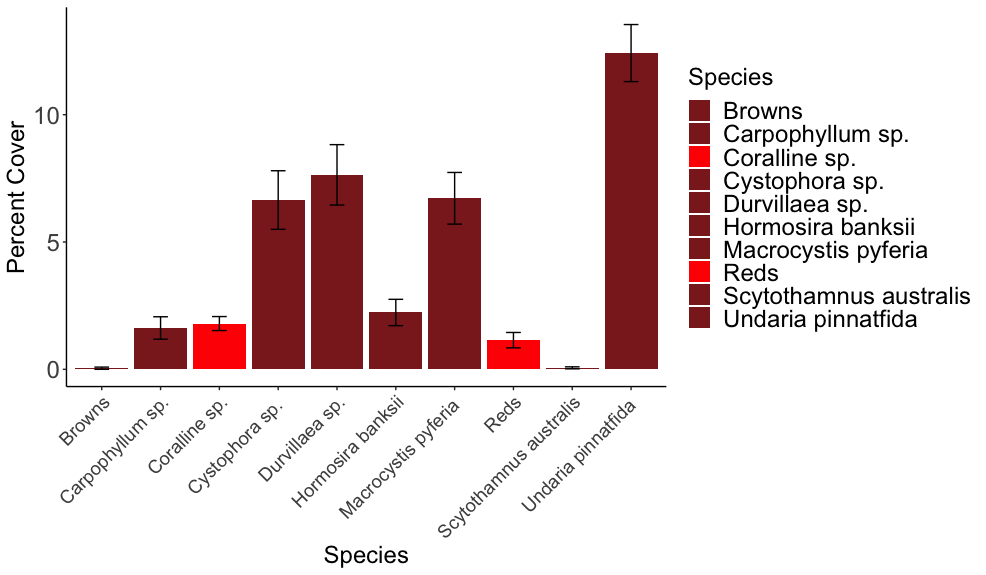


**Figure S2.** Average abundance (percent cover ± standard error) of the four most abundant sessile faunal taxonomic groups; bivalves, serpulidae tube worms, cnidaria and bryozoa before (2017, n = 12 1 m^2^ random plots, black points, grey shading, no elevation measurements) and after (n = 32 permanently marked 0.25 m^2^ plots, 2018-2022/23) the MHW, where post-MHW data were separated into low (blue) and mid (green) intertidal elevations during spring (circles) and autumn (triangles).

**
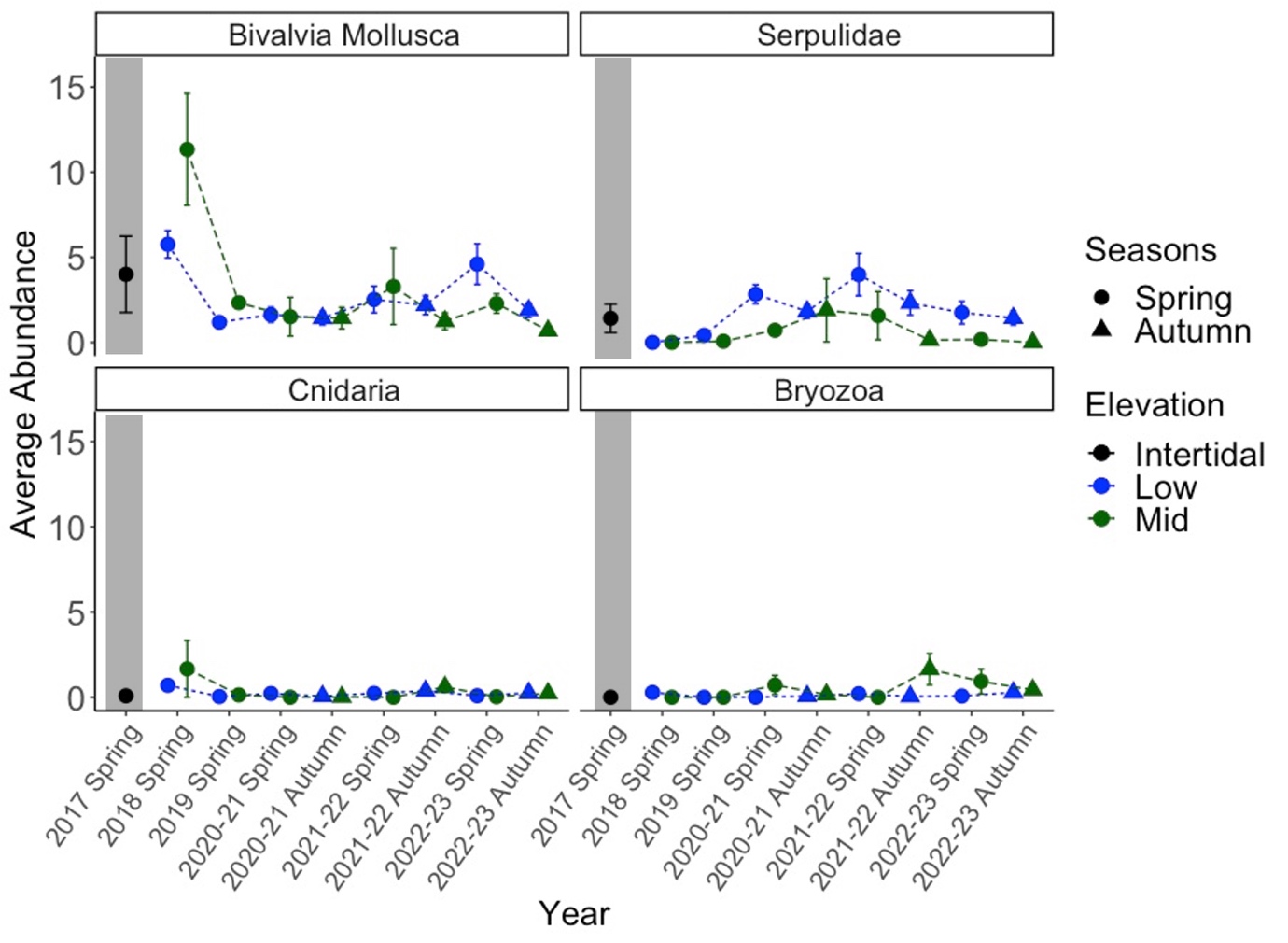
**

**Figure S3.** Average abundance (count ± standard error) of the three most abundant mobile taxonomic groups; mobile mollusca, crustacea and echinodermata before (2017, n = 12 1 m^2^ random plots, black points, grey shading, no elevation measurements) and after (n = 32 permanently marked 0.25 m^2^ plots, 2018-2022/23) the MHW, where post-MHW data were separated into low (blue) and mid (green) intertidal elevations during spring (circles) and autumn (triangles).

**
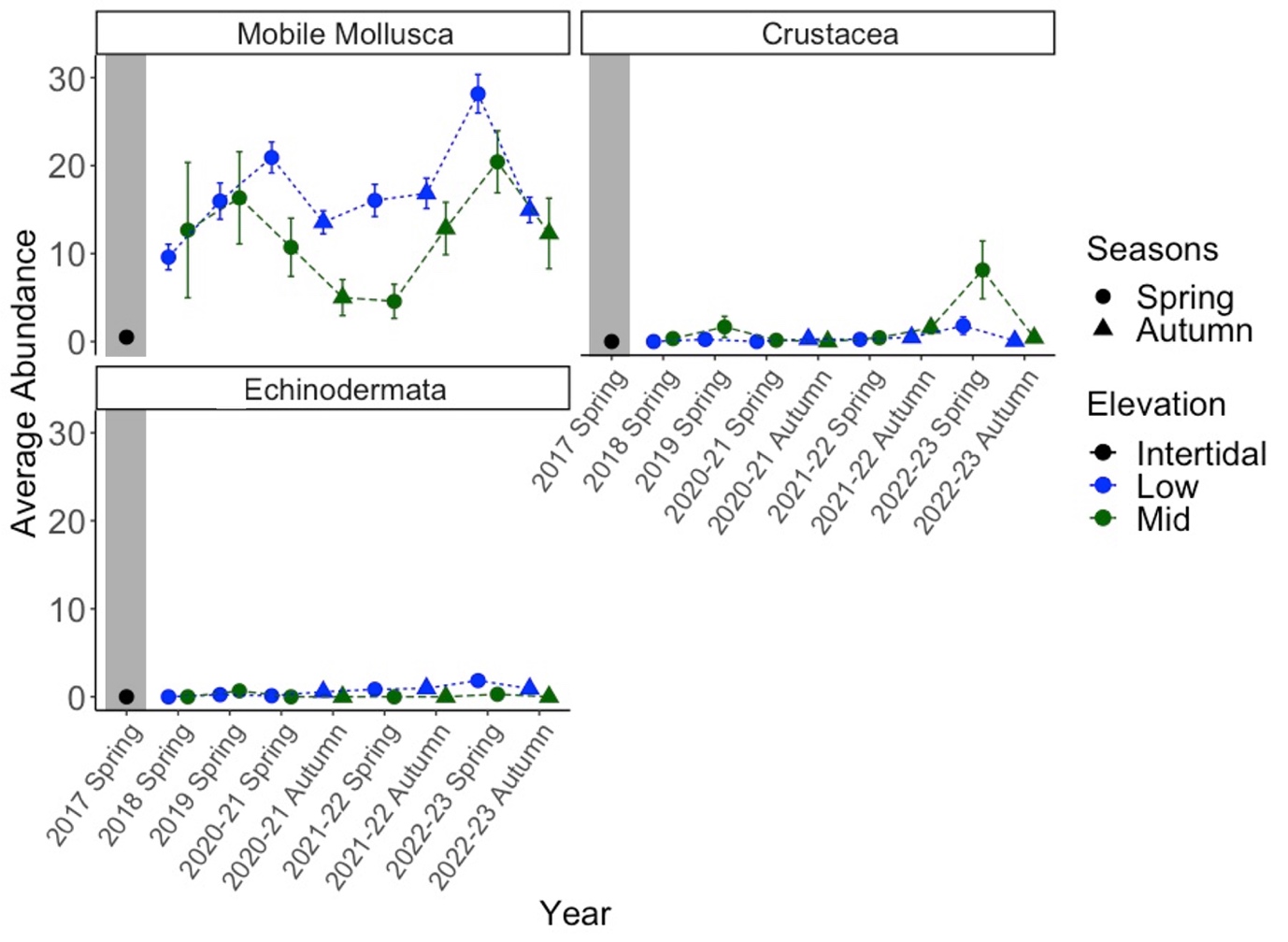
**

**Figure S4.** Average abundance (percent cover ± standard error) of dominant seaweed groups including fucales, green algae, laminariales, other browns, reds and scytothamnales (mainly *Splachnidium rugosum* and *Scytothamnus australis*) before (2017, n = 12 1 m^2^ random plots, black points, grey shading, no elevation measurements) and after (n = 32 permanently marked 0.25 m^2^ plots, 2018-2022/23) the MHW, where post-MHW data were separated into low (blue) and mid (green) intertidal elevations during spring (circles) and autumn (triangles).

**
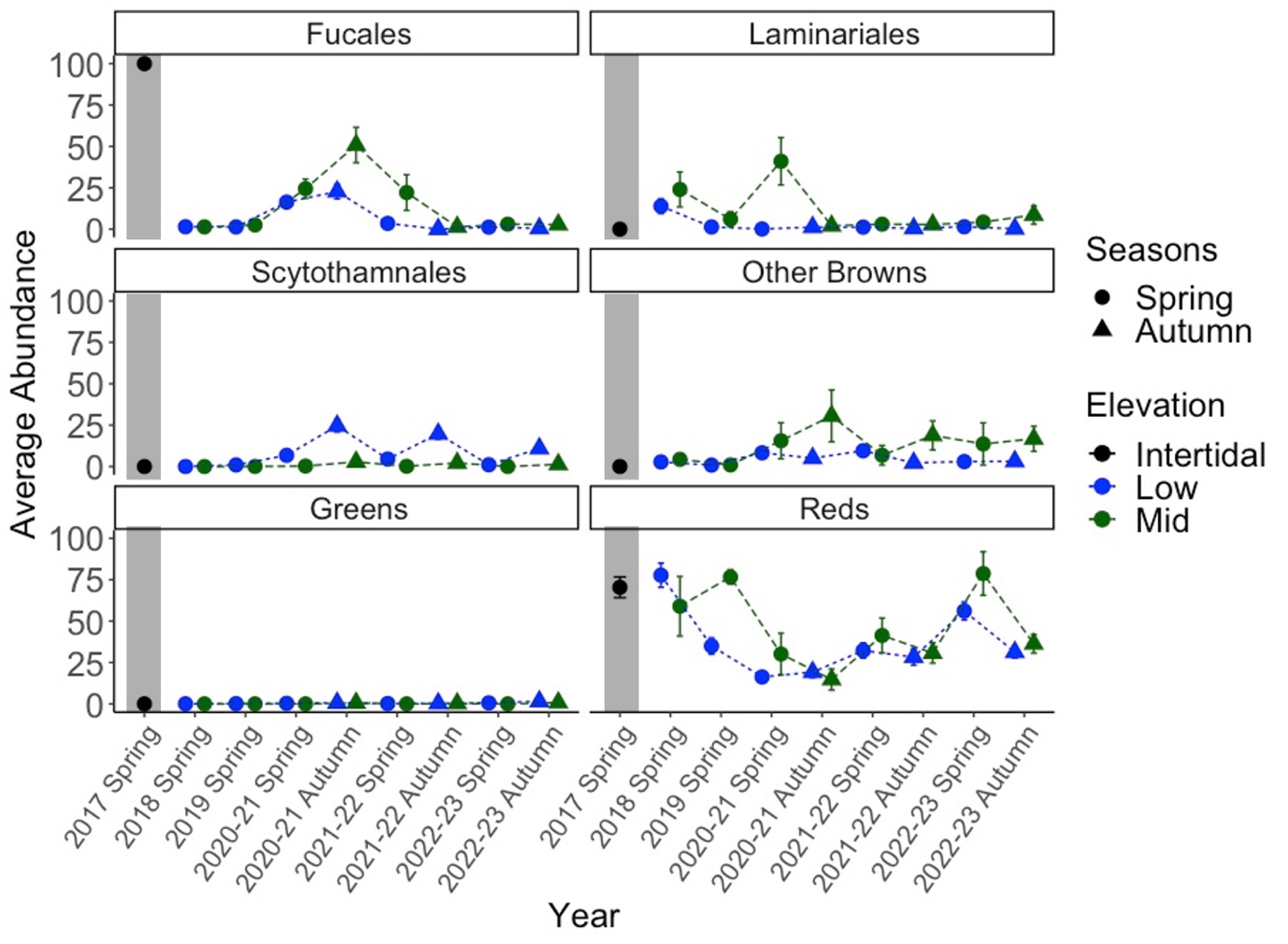
**

**Figure S5.** NMDS plot showing average community composition per year, from 2017 to 2022, and tidal elevation (low, mid, high) from drone scale annual surveys (top) and permanent plots during seasonal surveys in autumn and spring from 2020-21 to 2022-23 (bottom).


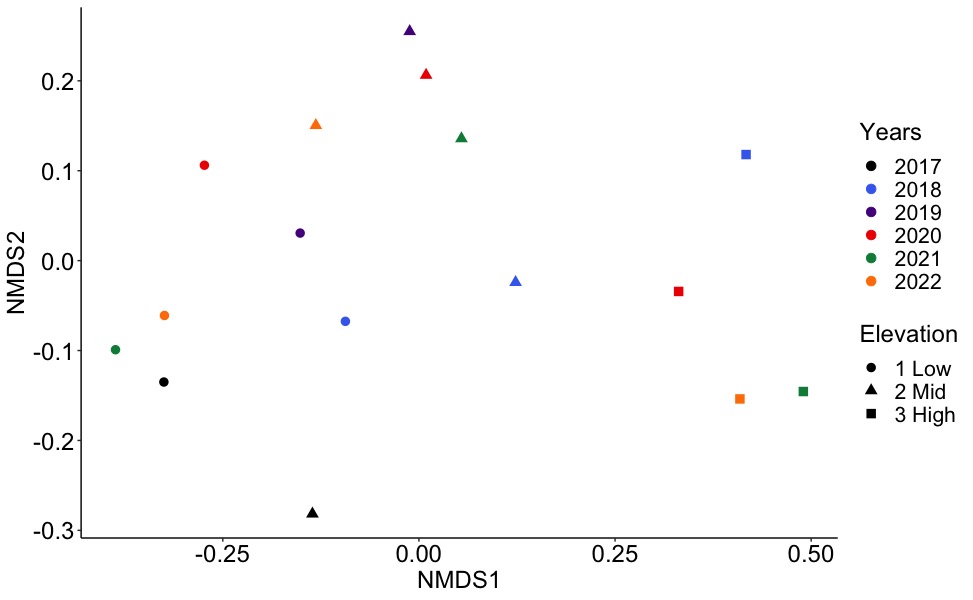


**
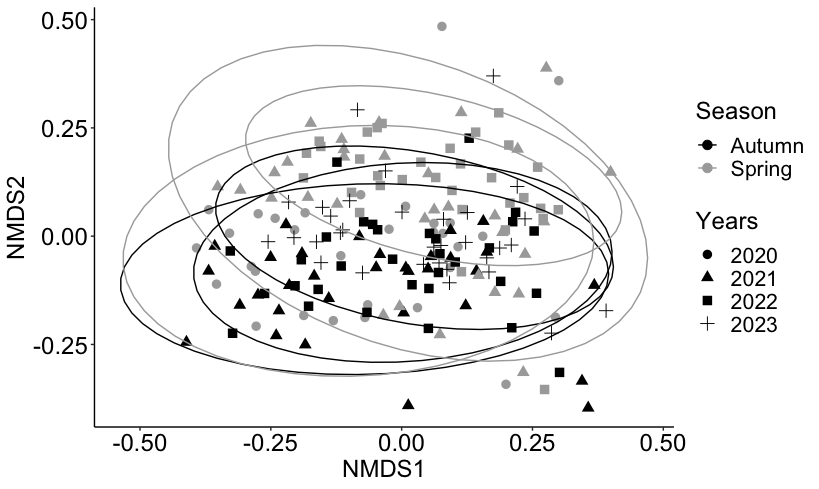
**

**Figure S7.** Attachment network showing all documented interactions permanent between host and epibiota with outgoing arrows/ links during spring low intertidal (A), spring mid intertidal (B), autumn low intertidal (C) and autumn mid intertidal zones (D).

**
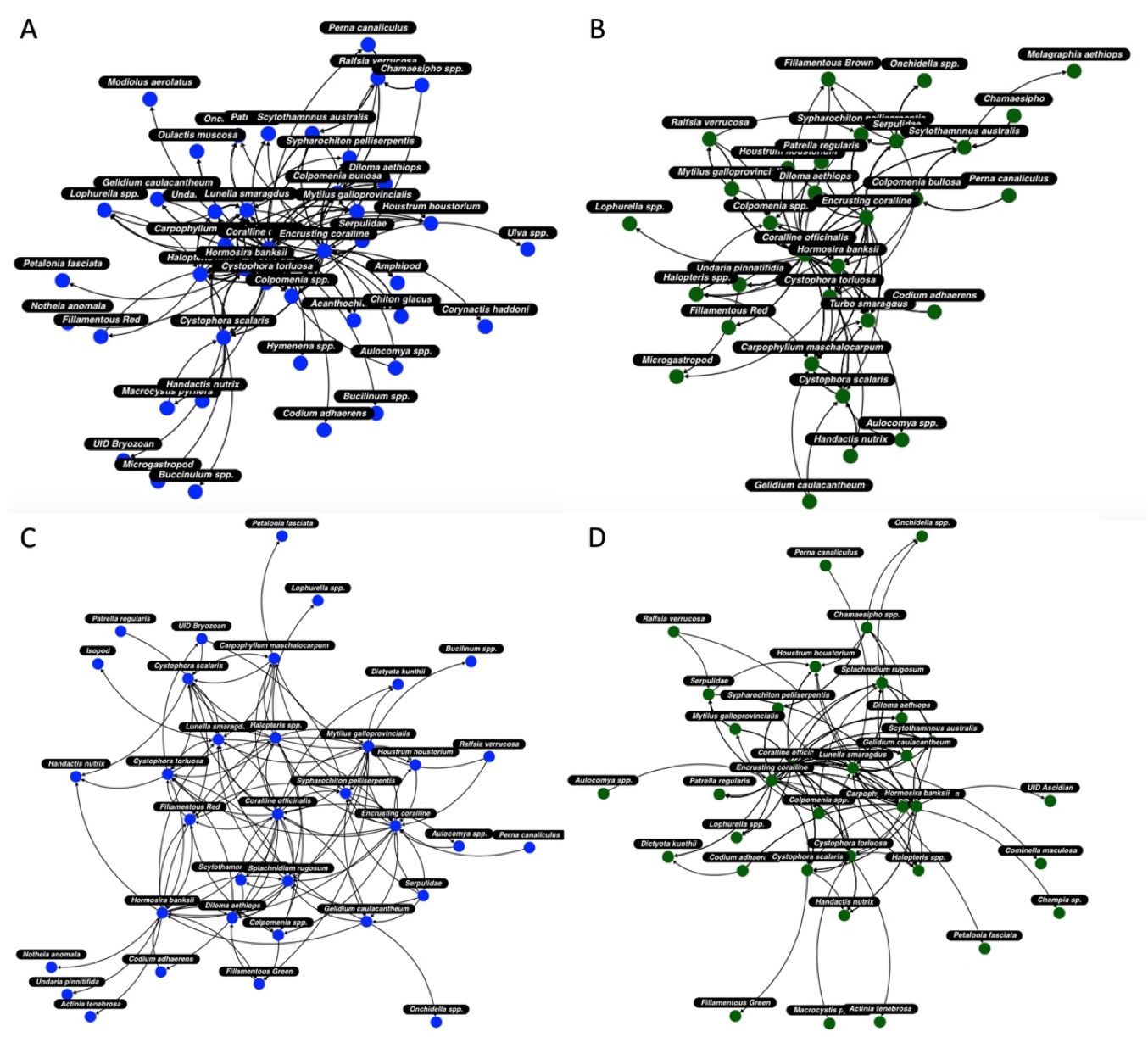
**

# **Tables**

**Table S1.** Repeated measures four-factorial Permutational (999 permutation) multivariate analysis of variance on percent cover of five key habitat forming seaweed species within permanent plots surveyed seasonally in autumn and spring after the MHW, between 2020-21 and 2022-23, using Euclidian distance for univariate responses (A) *H. banksii*, (B) *Undaria*, (C) *C. torulosa*, (D) *C. scalaris*, (E) *C. officinalis* and (F) encrusting coralline. Factors year (2018, 2019, 2020, 2021, 2022) and season (autumn, spring) were fixed and factors Undaria removal (control, removal) and elevation (low, mid) were nested within the random factor plot (a repeated measure). Significant p-values (alpha < 0.05) are shown on bold and p-values <0.1 are shown in brackets.

| **A. *H. banksii*** | Df | SS | % SS | Pseudo-F | P-Value |
| --- | --- | --- | --- | --- | --- |
| *Undaria* removal | 1 | 258.68 | 0.30 | 0.14 | 0.742 |
| Elevation | 1 | 2582.60 | 2.96 | 1.35 | 0.253 |
| Season | 1 | 2680.40 | 3.07 | 25.04 | **0.001** |
| Year | 2 | 1241.00 | 1.42 | 11.29 | **0.001** |
| Elevation x *Undaria* removal | 1 | 824.37 | 0.94 | 0.43 | 0.486 |
| *Undaria* removal x Season | 1 | 11.83 | 0.01 | 0.11 | 0.744 |
| *Undaria* removal x Year | 2 | 95.55 | 0.11 | 0.87 | 0.439 |
| Elevation x Season | 1 | 83.34 | 0.10 | 0.78 | 0.358 |
| Elevation x Year | 2 | 733.60 | 0.84 | 6.67 | **0.005** |
| Season x Year | 2 | 61.24 | 0.07 | 0.27 | 0.762 |
| Plot(*Undaria* removal x Elevation) | 29 | 63024.00 | 72.24 | 19.22 | **0.001** |
| *Undaria* x Elevation x Season | 1 | 281.40 | 0.32 | 2.63 | (0.099) |
| *Undaria* removal x Elevation x Year | 2 | 620.83 | 0.71 | 5.65 | **0.005** |
| *Undaria* removal x Season x Year | 2 | 205.31 | 0.24 | 0.91 | 0.411 |
| Elevation x Season x Year | 2 | 209.38 | 0.24 | 0.93 | 0.400 |
| Season x Plot(*Undaria* removal x Elevation) | 28 | 2996.80 | 3.43 | 0.95 | 0.539 |
| Year x Plot(*Undaria* removal x Elevation) | 56 | 3063.60 | 3.51 | 0.48 | 0.993 |
| *Undaria* removal x Elevation x Season x Year | 2 | 102.20 | 0.12 | 0.45 | 0.624 |
| Plot x Year x Season x Elevation x *Undaria* removal | 55 | 6217.70 | 7.13 | No Test | No Test |
| **B. *Undaria*** | Df | SS | % SS | Pseudo-F | P-Value |
| *Undaria* removal | 1 | 30.23 | 0.07 | 0.09 | 0.785 |
| Elevation | 1 | 1605.70 | 3.70 | 4.67 | **0.024** |
| Season | 1 | 1816.30 | 4.19 | 9.30 | **0.008** |
| Year | 2 | 689.22 | 1.59 | 2.70 | (0.093) |
| Elevation x *Undaria* removal | 1 | 105.71 | 0.24 | 0.31 | 0.636 |
| *Undaria* removal x Season | 1 | 42.88 | 0.10 | 0.22 | 0.645 |
| *Undaria* removal x Year | 2 | 55.95 | 0.13 | 0.22 | 0.797 |
| Elevation x Season | 1 | 750.89 | 1.73 | 3.85 | (0.066) |
| Elevation x Year | 2 | 215.36 | 0.50 | 0.85 | 0.444 |
| Season x Year | 2 | 1400.80 | 3.23 | 3.47 | **0.032** |
| Plot(*Undaria* removal x Elevation) | 29 | 10572.00 | 24.37 | 1.81 | **0.044** |
| *Undaria* x Elevation x Season | 1 | 176.31 | 0.41 | 0.90 | 0.365 |
| *Undaria* removal x Elevation x Year | 2 | 87.97 | 0.20 | 0.35 | 0.731 |
| *Undaria* removal x Season x Year | 2 | 158.18 | 0.36 | 0.39 | 0.678 |
| Elevation x Season x Year | 2 | 577.97 | 1.33 | 1.43 | 0.258 |
| Season x Plot(*Undaria* removal x Elevation) | 28 | 5467.70 | 12.60 | 0.97 | 0.513 |
| Year x Plot(*Undaria* removal x Elevation) | 56 | 7116.00 | 16.40 | 0.63 | 0.958 |
| *Undaria* removal x Elevation x Season x Year | 2 | 117.35 | 0.27 | 0.29 | 0.739 |
| Plot x Year x Season x Elevation x *Undaria* removal | 55 | 11090.00 | 25.56 | No Test | No Test |
| **C. *C. torulosa*** | Df | SS | % SS | Pseudo-F | P-Value |
| *Undaria* removal | 1 | 16.53 | 0.09 | 0.05 | 0.820 |
| Elevation | 1 | 157.17 | 0.84 | 0.51 | 0.503 |
| Season | 1 | 71.63 | 0.38 | 5.91 | **0.013** |
| Year | 2 | 1878.60 | 10.05 | 13.34 | **0.001** |
| Elevation x *Undaria* removal | 1 | 3.46 | 0.02 | 0.01 | 0.921 |
| *Undaria* removal x Season | 1 | 0.02 | 0.00 | 0.00 | 0.975 |
| *Undaria* removal x Year | 2 | 54.30 | 0.29 | 0.39 | 0.699 |
| Elevation x Season | 1 | 0.94 | 0.01 | 0.08 | 0.810 |
| Elevation x Year | 2 | 66.37 | 0.36 | 0.47 | 0.631 |
| Season x Year | 2 | 84.44 | 0.45 | 1.52 | 0.236 |
| Plot(*Undaria* removal x Elevation) | 29 | 10118.00 | 54.14 | 12.54 | **0.001** |
| *Undaria* x Elevation x Season | 1 | 7.83 | 0.04 | 0.65 | 0.436 |
| *Undaria* removal x Elevation x Year | 2 | 16.53 | 0.09 | 0.12 | 0.889 |
| *Undaria* removal x Season x Year | 2 | 14.87 | 0.08 | 0.27 | 0.780 |
| Elevation x Season x Year | 2 | 10.61 | 0.06 | 0.19 | 0.844 |
| Season x Plot(*Undaria* removal x Elevation) | 28 | 338.70 | 1.81 | 0.43 | 0.989 |
| Year x Plot(*Undaria* removal x Elevation) | 56 | 3953.20 | 21.15 | 2.54 | **0.001** |
| *Undaria* removal x Elevation x Season x Year | 2 | 46.21 | 0.25 | 0.83 | 0.444 |
| Plot x Year x Season x Elevation x *Undaria* removal | 55 | 1530.70 | 8.19 | No Test | No Test |
| **D. *C. scalaris*** | Df | SS | % SS | Pseudo-F | P-Value |
| *Undaria* removal | 1 | 36.91 | 0.70 | 0.42 | 0.548 |
| Elevation | 1 | 1.83 | 0.03 | 0.02 | 0.894 |
| Season | 1 | 129.52 | 2.44 | 18.89 | **0.001** |
| Year | 2 | 214.69 | 4.05 | 10.09 | **0.001** |
| Elevation x *Undaria* removal | 1 | 393.48 | 7.42 | 4.52 | **0.032** |
| *Undaria* removal x Season | 1 | 1.46 | 0.03 | 0.21 | 0.661 |
| *Undaria* removal x Year | 2 | 19.40 | 0.37 | 0.91 | 0.401 |
| Elevation x Season | 1 | 15.49 | 0.29 | 2.26 | 0.135 |
| Elevation x Year | 2 | 5.52 | 0.10 | 0.26 | 0.766 |
| Season x Year | 2 | 81.37 | 1.54 | 3.27 | **0.037** |
| Plot(*Undaria* removal x Elevation) | 29 | 2846.80 | 53.71 | 7.90 | **0.001** |
| *Undaria* x Elevation x Season | 1 | 0.39 | 0.01 | 0.06 | 0.835 |
| *Undaria* removal x Elevation x Year | 2 | 27.22 | 0.51 | 1.28 | 0.265 |
| *Undaria* removal x Season x Year | 2 | 6.67 | 0.13 | 0.27 | 0.783 |
| Elevation x Season x Year | 2 | 8.30 | 0.16 | 0.33 | 0.712 |
| Season x Plot(*Undaria* removal x Elevation) | 28 | 191.72 | 3.62 | 0.55 | 0.963 |
| Year x Plot(*Undaria* removal x Elevation) | 56 | 595.29 | 11.23 | 0.86 | 0.755 |
| *Undaria* removal x Elevation x Season x Year | 2 | 21.18 | 0.40 | 0.85 | 0.464 |
| Plot x Year x Season x Elevation x *Undaria* removal | 55 | 683.58 | 12.90 | No Test | No Test |
| **E. *C. officinalis*** | Df | SS | % SS | Pseudo-F | P-Value |
| *Undaria* removal | 1 | 42.07 | 0.04 | 0.04 | 0.846 |
| Elevation | 1 | 1153.30 | 1.14 | 1.00 | 0.340 |
| Season | 1 | 2783.10 | 2.76 | 7.56 | **0.010** |
| Year | 2 | 10441.00 | 10.35 | 18.11 | **0.001** |
| Elevation x *Undaria* removal | 1 | 536.67 | 0.53 | 0.46 | 0.526 |
| *Undaria* removal x Season | 1 | 99.73 | 0.10 | 0.27 | 0.638 |
| *Undaria* removal x Year | 2 | 27.27 | 0.03 | 0.05 | 0.954 |
| Elevation x Season | 1 | 668.33 | 0.66 | 1.82 | 0.188 |
| Elevation x Year | 2 | 653.95 | 0.65 | 1.13 | 0.337 |
| Season x Year | 2 | 5568.70 | 5.52 | 12.23 | **0.001** |
| Plot(*Undaria* removal x Elevation) | 29 | 37603.00 | 37.27 | 5.70 | **0.001** |
| *Undaria* x Elevation x Season | 1 | 28.66 | 0.03 | 0.08 | 0.802 |
| *Undaria* removal x Elevation x Year | 2 | 672.49 | 0.67 | 1.17 | 0.298 |
| *Undaria* removal x Season x Year | 2 | 218.33 | 0.22 | 0.48 | 0.629 |
| Elevation x Season x Year | 2 | 333.08 | 0.33 | 0.73 | 0.470 |
| Season x Plot(*Undaria* removal x Elevation) | 28 | 10312.00 | 10.22 | 1.62 | (0.060) |
| Year x Plot(*Undaria* removal x Elevation) | 56 | 16159.00 | 16.02 | 1.27 | 0.185 |
| *Undaria* removal x Elevation x Season x Year | 2 | 72.60 | 0.07 | 0.16 | 0.860 |
| Plot x Year x Season x Elevation x *Undaria* removal | 55 | 12518.00 | 12.41 | No Test | No Test |
| **F. Encrusting Coralline** | Df | SS | % SS | Pseudo-F | P-Value |
| *Undaria* removal | 1 | 10.01 | 0.04 | 0.04 | 0.820 |
| Elevation | 1 | 1.88 | 0.01 | 0.01 | 0.941 |
| Season | 1 | 1570.90 | 5.97 | 21.72 | **0.001** |
| Year | 2 | 1852.90 | 7.04 | 9.82 | **0.001** |
| Elevation x *Undaria* removal | 1 | 0.08 | 0.00 | 0.00 | 0.979 |
| *Undaria* removal x Season | 1 | 0.09 | 0.00 | 0.00 | 0.978 |
| *Undaria* removal x Year | 2 | 19.32 | 0.07 | 0.10 | 0.907 |
| Elevation x Season | 1 | 2.50 | 0.01 | 0.03 | 0.865 |
| Elevation x Year | 2 | 103.05 | 0.39 | 0.55 | 0.596 |
| Season x Year | 2 | 851.15 | 3.23 | 5.20 | **0.009** |
| Plot(*Undaria* removal x Elevation) | 29 | 9117.30 | 34.63 | 3.84 | **0.001** |
| *Undaria* x Elevation x Season | 1 | 38.09 | 0.14 | 0.53 | 0.459 |
| *Undaria* removal x Elevation x Year | 2 | 154.68 | 0.59 | 0.82 | 0.452 |
| *Undaria* removal x Season x Year | 2 | 350.61 | 1.33 | 2.14 | 0.146 |
| Elevation x Season x Year | 2 | 135.83 | 0.52 | 0.83 | 0.442 |
| Season x Plot(*Undaria* removal x Elevation) | 28 | 2025.10 | 7.69 | 0.88 | 0.644 |
| Year x Plot(*Undaria* removal x Elevation) | 56 | 5287.00 | 20.08 | 1.15 | 0.299 |
| *Undaria* removal x Elevation x Season x Year | 2 | 63.82 | 0.24 | 0.39 | 0.696 |
| Plot x Year x Season x Elevation x *Undaria* removal | 55 | 4505.20 | 17.11 | No Test | No Test |
|  |  |  |  |  |  |

**Table S2.** Four-factorial Permutational multivariate analysis of variance on community structure, using Bray-Curtis dissimilarity coefficients for multivariate data, within permanent plots surveyed seasonally in autumn and spring, between 2020-21 and 2022-23.

| **Community** | Df | SS | % SS | F.Model | R2 | P-value |
| --- | --- | --- | --- | --- | --- | --- |
| *Undaria* removal | 1 | 0.24 | 0.63 | 1.36 | 0.006 | 0.175 |
| Elevation | 1 | 1.06 | 2.83 | 6.08 | 0.028 | **0.001** |
| Year | 2 | 2.31 | 6.18 | 6.62 | 0.062 | **0.001** |
| Season | 1 | 2.17 | 5.81 | 12.47 | 0.058 | **0.001** |
| Elevation x *Undaria* removal | 1 | 0.36 | 0.97 | 2.07 | 0.010 | **0.028** |
| Year x *Undaria* removal | 2 | 0.06 | 0.17 | 0.18 | 0.002 | 1.000 |
| Year x Elevation | 2 | 0.27 | 0.72 | 0.77 | 0.007 | 0.744 |
| Year x Season | 2 | 0.99 | 2.66 | 2.85 | 0.027 | **0.001** |
| *Undaria* removal x Season | 1 | 0.03 | 0.09 | 0.19 | 0.001 | 0.998 |
| Elevation x Season | 1 | 0.13 | 0.35 | 0.74 | 0.003 | 0.695 |
| Year x Elevation x *Undaria* removal | 2 | 0.07 | 0.18 | 0.19 | 0.002 | 1.000 |
| Year x Elevation x Season | 2 | 0.15 | 0.39 | 0.42 | 0.004 | 0.993 |
| Year x *Undaria* removal x Season | 2 | 0.11 | 0.29 | 0.31 | 0.003 | 1.000 |
| Elevation x *Undaria* removal x Season | 1 | 0.09 | 0.24 | 0.50 | 0.002 | 0.909 |
| Year x Elevation x *Undaria* removal x Season | 2 | 0.07 | 0.18 | 0.20 | 0.002 | 1.000 |
| Residuals | 168 | 29.26 | 78.32 | 0.78 |  |  |
| Total | 191 | 37.36 |  |  |  |  |

**Table S3.** Repeated measures four-factorial Permutational (999 permutations) multivariate analysis of variance on percent cover of sessile species and counts of mobile species within permanent plots surveyed seasonally in autumn and spring after the MHW, between 2020-21 and 2022-23, using Euclidian distance for univariate responses for (A) sessile richness, (B) mobile richness, (C) sessile abundance and (D) mobile abundance. Factors year (2018, 2019, 2020, 2021, 2022) and season (autumn, spring) were fixed and factors *Undaria* removal (control, removal) and elevation (low, mid) were nested within the random factor plot (a repeated measure). Significant p-values (alpha < 0.05) are shown on bold and p-values <0.1 are shown in brackets.

| **A. Sessile Richness** | Df | SS | % SS | Pseudo-F | P-Value |
| --- | --- | --- | --- | --- | --- |
| *Undaria* removal | 1 | 68.35 | 0.28 | 0.32 | 0.589 |
| Elevation | 1 | 220.75 | 0.90 | 1.02 | 0.362 |
| Season | 1 | 1825 | 7.47 | 26.42 | **0.001** |
| Year | 2 | 2322.10 | 9.51 | 20.35 | **0.001** |
| Elevation x *Undaria* removal | 1 | 213.77 | 0.88 | 0.99 | 0.362 |
| *Undaria* removal x Season | 1 | 64.55 | 0.26 | 0.93 | 0.309 |
| *Undaria* removal x Year | 2 | 93.58 | 0.38 | 0.82 | 0.462 |
| Elevation x Season | 1 | 5.03 | 0.02 | 0.07 | 0.764 |
| Elevations x Year | 2 | 288.09 | 1.18 | 2.52 | (0.080) |
| Season x Year | 2 | 2559.10 | 10.48 | 21.14 | **0.001** |
| Plot(*Undaria* removal x Elevation) | 29 | 6957.40 | 28.50 | 3.96 | **0.001** |
| *Undaria* x Elevation x Season | 1 | 35.06 | 0.14 | 0.51 | 0.468 |
| *Undaria* removal x Elevation x Year | 2 | 2.79 | 0.01 | 0.02 | 0.979 |
| *Undaria* removal x Season x Year | 2 | 139.34 | 0.57 | 1.15 | 0.309 |
| Elevation x Season x Year | 2 | 91.82 | 0.38 | 0.76 | 0.485 |
| Season x Plot(*Undaria* removal x Elevation) | 28 | 1934.50 | 7.92 | 1.14 | 0.357 |
| Year x Plot(*Undaria* removal x Elevation) | 56 | 3194.40 | 13.08 | 0.94 | 0.586 |
| *Undaria* removal x Elevation x Season x Year | 2 | 52.66 | 0.22 | 0.44 | 0.638 |
| Plot x Year x Season x Elevation x *Undaria* removal | 55 | 3328.70 | 13.63 | No Test | No Test |
| **B. Mobile Richness** | Df | SS | % SS | Pseudo-F | P-Value |
| *Undaria* removal | 1 | 0.44 | 0.07 | 0.06 | 0.819 |
| Elevation | 1 | 6.13 | 0.94 | 0.85 | 0.360 |
| Season | 1 | 1.74 | 0.27 | 0.96 | 0.348 |
| Year | 2 | 33.09 | 5.08 | 8.73 | **0.001** |
| Elevation x *Undaria* removal | 1 | 3.67 | 0.56 | 0.51 | 0.466 |
| *Undaria* removal x Season | 1 | 0 | 0 | 0 | 0.974 |
| *Undaria* removal x Year | 2 | 4.74 | 0.73 | 1.25 | 0.315 |
| Elevation x Season | 1 | 0.45 | 0.07 | 0.25 | 0.648 |
| Elevations x Year | 2 | 0.94 | 0.14 | 0.25 | 0.771 |
| Season x Year | 2 | 8.23 | 1.26 | 1.36 | 0.240 |
| Plot(*Undaria* removal x Elevation) | 29 | 226.87 | 34.80 | 2.58 | **0.002** |
| *Undaria* x Elevation x Season | 1 | 1.02 | 0.16 | 0.56 | 0.456 |
| *Undaria* removal x Elevation x Year | 2 | 9.74 | 1.49 | 2.57 | (0.090) |
| *Undaria* removal x Season x Year | 2 | 7.28 | 1.12 | 1.20 | 0.335 |
| Elevation x Season x Year | 2 | 1.04 | 0.16 | 0.17 | 0.837 |
| Season x Plot(*Undaria* removal x Elevation) | 28 | 50.87 | 7.80 | 0.60 | 0.934 |
| Year x Plot(*Undaria* removal x Elevation) | 56 | 105.88 | 16.24 | 0.62 | 0.961 |
| *Undaria* removal x Elevation x Season x Year | 2 | 9.46 | 1.45 | 1.56 | 0.234 |
| Plot x Year x Season x Elevation x *Undaria* removal | 55 | 166.61 | 25.55 | No Test | No Test |
| **C. Sessile Abundance** | Df | SS | % SS | Pseudo-F | P-Value |
| *Undaria* removal | 1 | 503.24 | 0.18 | 0.11 | 0.723 |
| Elevation | 1 | 2.81 | 0 | 0 | 0.984 |
| Season | 1 | 284.30 | 0.10 | 0.38 | 0.547 |
| Year | 2 | 26426 | 9.58 | 21.15 | **0.001** |
| Elevation x *Undaria* removal | 1 | 3623.70 | 1.31 | 0.80 | 0.356 |
| *Undaria* removal x Season | 1 | 0 | 0 | 0 | 1.000 |
| *Undaria* removal x Year | 2 | 14.04 | 0.01 | 0.01 | 0.984 |
| Elevation x Season | 1 | 524.42 | 0.19 | 0.70 | 0.418 |
| Elevations x Year | 2 | 2543.50 | 0.92 | 2.04 | 0.135 |
| Season x Year | 2 | 6874 | 2.49 | 7.06 | **0.001** |
| Plot(*Undaria* removal x Elevation) | 29 | 149020 | 54.03 | 10.56 | **0.001** |
| *Undaria* x Elevation x Season | 1 | 1.13 | 0 | 0 | 0.975 |
| *Undaria* removal x Elevation x Year | 2 | 247.12 | 0.09 | 0.20 | 0.824 |
| *Undaria* removal x Season x Year | 2 | 547.45 | 0.20 | 0.56 | 0.572 |
| Elevation x Season x Year | 2 | 887.10 | 0.32 | 0.91 | 0.417 |
| Season x Plot(*Undaria* removal x Elevation) | 28 | 20957 | 7.60 | 1.54 | (0.096) |
| Year x Plot(*Undaria* removal x Elevation) | 56 | 35022 | 12.70 | 1.28 | 0.163 |
| *Undaria* removal x Elevation x Season x Year | 2 | 368.66 | 0.13 | 0.38 | 0.655 |
| Plot x Year x Season x Elevation x *Undaria* removal | 55 | 26771 | 9.71 | No Test | No Test |
| **D. Mobile Abundance** | Df | SS | % SS | Pseudo-F | P-Value |
| *Undaria* removal | 1 | 2.02 | 0.14 | 0.10 | 0.744 |
| Elevation | 1 | 19.13 | 1.30 | 0.92 | 0.355 |
| Season | 1 | 46.65 | 3.16 | 10.21 | **0.007** |
| Year | 2 | 26.51 | 1.79 | 2.81 | (0.069) |
| Elevation x *Undaria* removal | 1 | 3.24 | 0.22 | 0.16 | 0.695 |
| *Undaria* removal x Season | 1 | 1.34 | 0.09 | 0.29 | 0.581 |
| *Undaria* removal x Year | 2 | 9.50 | 0.64 | 1.01 | 0.359 |
| Elevation x Season | 1 | 2.63 | 0.18 | 0.58 | 0.468 |
| Elevations x Year | 2 | 22.82 | 1.54 | 2.42 | (0.098) |
| Season x Year | 2 | 46.66 | 3.16 | 6.50 | **0.001** |
| Plot(*Undaria* removal x Elevation) | 29 | 676.97 | 45.83 | 6.50 | **0.001** |
| *Undaria* x Elevation x Season | 1 | 7.75 | 0.52 | 1.70 | 0.227 |
| *Undaria* removal x Elevation x Year | 2 | 3.45 | 0.23 | 0.37 | 0.681 |
| *Undaria* removal x Season x Year | 2 | 10.21 | 0.69 | 1.42 | 0.265 |
| Elevation x Season x Year | 2 | 7.27 | 0.49 | 1.01 | 0.362 |
| Season x Plot(*Undaria* removal x Elevation) | 28 | 127.99 | 8.66 | 1.27 | 0.227 |
| Year x Plot(*Undaria* removal x Elevation) | 56 | 264.66 | 17.92 | 1.32 | 0.136 |
| *Undaria* removal x Elevation x Season x Year | 2 | 13.79 | 0.93 | 1.92 | 0.139 |
| Plot x Year x Season x Elevation x *Undaria* removal | 55 | 197.52 | 13.37 | No Test | No Test |
|  |  |  |  |  |  |
